# Creative arts-based interventions for the improvement of PTSD symptoms in young people: a meta-analysis with a focus on non-Western populations

**DOI:** 10.1038/s44220-025-00543-y

**Published:** 2025-11-27

**Authors:** Briana Applewhite, Brennan Delattre, Ilina Singh, Morten Kringelbach, Olivia Spiegler

**Affiliations:** 1 Department of Psychiatry, University of Oxford, Oxford, UK; 2 Centre for Eudaimonia and Human Flourishing, Oxford, UK; 3 Oxford Health NHS Foundation Trust, Oxford, UK; 4 Aarhus University, Aarhus, Denmark; 5 Department of Sociology, University of Oxford, Oxford, UK; 6 PFH Private University of Applied Sciences, Gottingen, Germany

**Keywords:** Psychology, Psychology

## Abstract

Post-traumatic stress disorder (PTSD) is increasingly prevalent among young people, yet current evidence-based treatments show variable outcomes. Creative arts-based interventions (for example, music, dance, visual art and drama) are gaining attention as complementary approaches to trauma care. Here we evaluated the effectiveness of creative arts-based interventions in reducing PTSD symptoms among youth globally, with particular attention to underrepresented and non-Western populations. We searched PubMed, PsycINFO and Web of Science for studies published up to 16 June 2025. Eligible studies were randomized controlled trials and quasi-experimental studies evaluating creative arts-based interventions with participants aged 3–18 years with diagnosed PTSD or trauma-related symptoms and validated pre-post PTSD outcomes. A random-effects meta-analysis was conducted with subgroup analyses by region and trauma severity. Thirty-three studies (*N* = 4,587) met inclusion criteria. Creative arts-based interventions significantly reduced PTSD symptoms (Hedges’ *g* *=* 0.85, 95% CI = 0.70–1.00). Strong effects were observed among participants with diagnosed PTSD and general trauma symptoms. Subgroup analyses showed large effects in West African and Middle Eastern samples, but no significant effects in Western populations. Although regional evidence was limited and intervention heterogeneity may affect generalizability, findings highlight creative arts-based interventions as effective and culturally resonant tools for reducing PTSD symptoms in youth, particularly in non-Western contexts. Future research should prioritize culturally focused, high-quality studies to assess applicability across diverse settings. This study was registered in PROSPERO (CRD42023389789).

## Abstract (web summary)

Current psychotherapies for post-traumatic stress disorder (PTSD) in children and adolescents often fail, especially within non-Western and ethnic minority populations. In this meta-analysis, the authors consolidate the most recent evidence regarding creative arts-based interventions for youth suffering from PTSD, underscoring their effectiveness, particularly for non-Western populations such as West African and Middle Eastern youth, and stressing the necessity for more culturally tailored and high-quality randomized controlled trials.

## Main

Posttraumatic stress disorder (PTSD) is a chronic and debilitating mental health disorder that develops after exposure to a traumatic event^1^. According to recent estimates, approximately 320 million people or 3.9% of the world’s population have had PTSD at some point in their lifetime^2^. The reasons for such a high prevalence of PTSD point largely to direct or indirect exposure to traumatic incidences such as physical violence, sexual trauma, serious injuries, natural disasters and death^3^. It is estimated that 25% of children and adolescents around the world will experience a traumatic event before reaching adulthood^4–7^. In a US epidemiological study, the prevalence of PTSD in adolescents aged 13–17 years was 4.7% (ref. ^8^). PTSD can cause substantial distress in a child’s life and could result in functional disabilities that could impair social, academic and occupational functioning if these chronic traumas are left untreated^9^.

Current therapeutic strategies to treat PTSD in children and adolescents include trauma-focused cognitive behavioural therapy (tf-CBT), CBT for PTSD, group trauma-focused CBT and eye movement desensitisation and reprocessing (EMDR) for persistent PTSD symptoms^10^. Pharmacological interventions are not recommended for children and adolescents with PTSD^10^. Despite CBT and EMDR being offered for the alleviation of PTSD symptoms in children and adolescents, nonresponse to CBT in both individual and group settings has been reported to be as high as 50%^3,11^. Recent evidence for the use of EMDR has found no significant difference between EMDR and other psychological treatments such as CBT in reducing PTSD symptoms^12^, further highlighting the variability of treatment efficacy. While tf-CBT has been suggested to be effective in Western populations^13,14^, there is little research concerning its efficacy in non-Western populations. To address the unique language and cultural needs of these populations, culturally adapted CBT was created^15^ and preliminary evidence has shown culturally adapted CBT to be effective in refugee and asylum seeker populations with trauma^16^. In it, the main elements of CBT have been preserved; however, the language, metaphors, techniques and examples used in CBT have been adapted to be culturally specific^15^.

Despite attempts at culturally adapted talking therapies, ethnic minority groups in Western countries are less likely to access therapies for their psychiatric disorders^17^. They also are less likely to have good outcomes in therapy and are more likely to report negative experiences in therapy, compared to white majority service users^18–20^. Furthermore, psychotherapy for refugees and other vulnerable populations is scarce because of the high cost and limited skills among trauma specialists to meet the language and cultural needs of these populations^21^. This highlights the need for novel, accessible and effective treatment methods for underrepresented, non-Western populations experiencing trauma symptoms and PTSD^22,23^.

As noted in the arts and health glossary by Davies and Clift^24^, art therapies are a form of psychotherapy that involve a therapeutic relationship between a qualified therapist and an individual who engages in creative activities for diagnostic or remedial purposes^25^. Creative arts therapies (CATs) are treatment interventions that involve art activities within therapy sessions and delivered by a trained creative arts therapist^25^. Using creative arts therapeutically can either refer to trained therapists using creative arts methods in their sessions or community-based creative arts interventions administered by community-based creative arts instructors with no mental health or therapeutic training. The National Coalition of Creative Arts Therapies Associations (NCCATA) defines creative arts therapists as ‘human service professionals who use distinct arts-based methods and creative processes for the purpose of ameliorating disability and illness and optimizing health and wellness’^24^. For this analysis, we define CATs as music, visual art, dance movement, poetry and drama therapy facilitated by a trained creative arts therapist and creative arts-based interventions as any intervention utilizing music, movement, visual arts, poetry or drama with the interventions carried out by a trained facilitator.

Creative arts-based interventions may provide novel solutions and alternative options to existing psychotherapies with variable success. Specifically, creative arts are unique therapeutic modalities that have the potential to overcome language barriers by utilizing predominantly nonverbal modalities that allow for the emotional expression of complex feelings in a nonverbal format^21^. Creative arts-based interventions can also be viewed as a therapeutic strategy that may mitigate stigma surrounding mental health treatments^26^, as they are not traditional talking therapies, which have been implicated in lacking engagement specifically with minoritized populations. In traditional talking therapies, these patients have reported feeling unwelcomed in services and stigmatized^27,28^. In a scoping review of over 3,000 studies on the role of arts in improving health and well-being, Fancourt and Finn^29^ found that the arts play a large role in the prevention of ill health and promote good health^29^. Arts also have an array of benefits related to building self-esteem, self-acceptance, confidence and self-worth, which are all preventative characteristics of mental illness^29^.

For Western samples, CATs have been shown to be tentatively promising for reducing symptoms of depression^30^, anxiety and obsessive–compulsive disorder^31^, anorexia nervosa^32,33^ and autism spectrum disorder^34^. They further help with the psychotic symptoms found in schizophrenia^35^. Evidence for the effectiveness of creative arts-based interventions for non-Western individuals with mental health symptoms has been growing. Among 500 Turkish children, for example, depressive symptoms decreased after a group Turkish traditional folk-dancing intervention^36^. Additionally, in a group dance movement therapy and art therapy intervention with 470 participants, PTSD symptoms reduced in Nigerian children who endured a traumatic event^37^. In West African cultures, specifically Nigerian culture, music plays an indispensable role at work, in politics, in socioeconomic engagements, in religious worship and in moral life^38^. In the Yoruba tribes in Nigeria, music is reported to create a diasporic consciousness and becomes an important medium for maintaining contact and identity^39^. Together, these findings suggest that creative arts-based interventions might be an effective treatment in non-Western populations as they incorporate valued cultural elements.

Systematic reviews compiling the existing literature on the usage of creative arts-based interventions as effective treatment methods for children and adolescents with symptoms of trauma and PTSD have been largely inconclusive^40,41^. In these reviews, researchers compiled existing case studies, intervention studies and qualitative studies to assess the evidence for the use of creative arts-based interventions for PTSD in children, but owing to the heterogeneity of methods and outcome measurements utilized, it was difficult to make firm conclusions on these interventions’ effectiveness^40,41^. A recently published meta-analysis on the effectiveness of creative arts interventions for treating children and adolescents exposed to traumatic events found that creative arts interventions significantly reduced PTSD symptom scores compared to baseline scores (Hedges’ *g* of 0.67 and *P* < 0.001)^42^. However, the meta-analysis had significant limitations owing to the limited number of studies (15 pre–post and 7 with a control group), the heterogeneity of traumatic experience and intervention type, and the lack of exploration into the effectiveness of CATs in underrepresented children and adolescents.

The aims of the present research were twofold. First, we aimed to provide robust empirical evidence for the effectiveness of creative arts-based interventions among children and adolescents. To achieve this, the present meta-analysis includes only quantitative experimental studies, randomized controlled trials (RCTs) and pre–post intervention studies. We also examine which types of creative art intervention reduce PTSD scores and how well creative arts-based interventions work based on PTSD diagnostic status and the duration of the intervention. Second, we aimed to examine whether creative arts-based interventions are more effective among non-Western than Western youth.

## Results

### Study selection

**Fig. 1:**
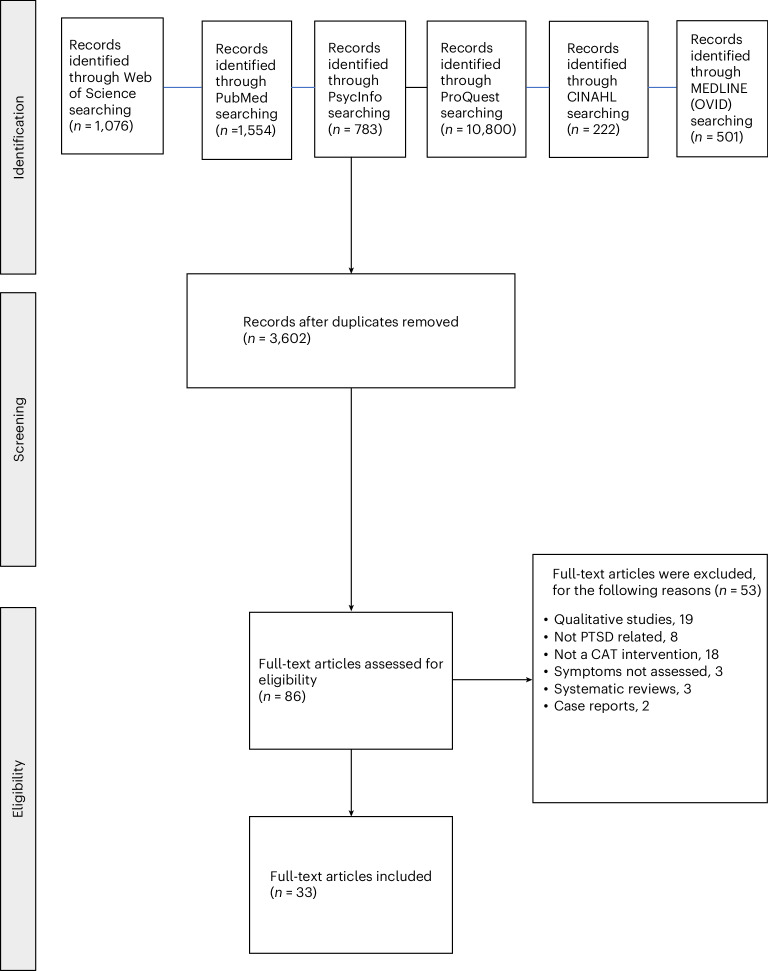
PRISMA Flow diagram of the screening and study selection process. PRISMA flow diagram illustrating the identification, screening and selection of studies (*n* = 33). Studies were published up to June 16, 2025. Source Data

A total of 3,602 records were screened after duplicates were removed. Overall, 86 full-text articles were assessed for eligibility, of which 53 were excluded for the following reasons: not a creative arts therapy (*n* = 18), not PTSD-related (*n* = 8), qualitative-only study (*n* = 19), symptoms not assessed (*n* = 3), systematic review (*n* = 3) and case report (*n* = 2). A total of 33 studies were included in the meta-analysis. The Preferred Reporting Items for Systematic Reviews and Meta-Analyses (PRISMA)^43^ flow chart is shown in Fig. 1.

The duration of the studies ranged from 5 days to 36 weeks (mean (*M*) = 10.06 weeks, s.d. of 6.88 weeks). The age of those allocated to creative arts-based interventions ranged from 3 to 18 years (*M* = 12.76 years, s.d. of 2.58 years). All studies were conducted on children and young people who had symptoms of trauma or a PTSD diagnosis according to the Diagnostic and Statistical Manual of Mental Disorders, 5th edition (DSM-5)^44^.

The type of creative arts-based intervention varied greatly. Studies utilized art, poetry, dance or dance movement-based interventions, music or music therapy as standalone treatments or a mixture of multiple creative interventions together. Table 1 provides a summary of the study and sample characteristics for each study.

**Table 1:** Summary of the studies included in the meta-analysis. Studies are listed in alphabetical order

Author(s), country	Sample size (*n*)	Study design	Control group	Control condition	Sessions	Duration (weeks)	PTSD measure	Region (nationality)	Type	Standalone modality versus combined	Mode of delivery	Delivered by	Setting	Diagnostic status
Anazor et al.^45^, Nigeria	400	Pre–post	Yes	No intervention	N.R	26	ITQ	Africa (Nigerian)	Art	Standalone	Group, interactive television	Licensed creative arts therapist	School	PTSD symptoms
Anichebe et al.^46^, Nigeria	342	RCT	Yes	TAU (unspecified)	12	6	ITQ	Africa (Nigerian)	Drama or music	Standalone	Group, in person	Licensed creative arts therapist	N.R.	Trauma symptoms
Bleile et al.^47^, Uganda	511	RCT	Yes	No intervention	11	N.R.	CRIES-8	Africa (Ugandan, South Sudanese)	Dance	Standalone	Group, in person	Trained facilitator	Refugee settlement	Trauma symptoms
Brillantes-Evangelista^48^, Philippines	29	Pre–post	No	N/A	8	8	CROPS	Asia (Filipino)	Art or poetry	Standalone	Group, in person	Trained facilitator	Hospital	PTSD symptoms
Burruss et al.^49^, USA	28	Pre–post	No	N/A	4–10	N.R.	SDQ	Mixed (mixed)	Art	Standalone	Group, in person	Licensed creative arts therapist	Community sites	Trauma symptoms
Colegrove et al.^50^, Australia	26	RCT	Yes	TAU (unspecified)	8	8	SDQ	Mixed (mixed)	Music	Standalone	Individual, in person	Licensed creative arts therapist	Community center	Trauma symptoms
Culver et al.^51^, Haiti	36	RCT	Yes	Waitlist control	N.R.	8	UCLA PTSD-RI	Caribbean (Haitian)	Dance	Standalone	Group, in person	Trained facilitator	School	Trauma symptoms
Dauber et al.^52^, USA	31	Pre–post	No	N/A	9–10	12	TSCC	North America (American)	Art	Standalone	Individual and group, in person	Licensed creative arts therapist	Outpatient center	Trauma symptoms
Decosimo et al.^53^, Nigeria	356	Pre–post	No	N/A	N.R	20	PSS	Africa (Nigerian)	Art, play, yoga	Combined	Group, in person	Licensed creative arts therapist	Sites within community	Trauma symptoms
Ewulu et al.^54^, Nigeria	323	Pre–post	Yes	No intervention	10	10	ITQ	Africa (Nigerian)	Music or art	Standalone	Group, in person with interactive media	Licensed creative arts therapist	N.R.	PTSD symptoms
Ezeh et al.^37^, Nigeria	470	RCT	Yes	No intervention	20	10	ITQ	Africa (Nigerian)	Dance or art	Standalone	Group, interactive media	Licensed creative arts therapist	N.R.	Trauma symptoms
Feen-Calligan et al.^55^, USA	24	Pre–post	Yes	No intervention	12	12	UCLA PTSD-RI	Middle East (Syrian)	Art	Standalone	Group, in person	Licensed creative arts therapist	Intervention site	PTSD symptoms
Gordon et al.^56^, USA	77	RCT	Yes	Waitlist control	12	6	HTQ	Europe (mixed)	Mix of creative expression	Combined	Group, in person	Trained facilitator	School	PTSD diagnosis
Grasser et al.^21^, USA	20	Pre–post	No	N/A	12	12	UCLA PTSD-RI	Middle East (Syrian)	Dance	Standalone	Group, in person	Licensed creative arts therapist	Community site	PTSD symptoms
Hylton et al.^57^, USA	34	Pre–post	No	N/A	N.R.	2	CRTES	North America (American)	Art, music, drama	Combined	Group, in person	Licensed creative arts therapist	School	PTSD diagnosis
Iyendo et al.^58^, Nigeria	429	RCT	Yes	No intervention	6	6	ITQ	Africa (Nigerian)	Art or music	Standalone	Group, interactive media	Licensed creative arts therapist	School	PTSD symptoms
Kalthom et al.^59^, Iran	20	RCT	Yes	No intervention	12	6	PCL-C	Middle East (Syrian)	Art	Standalone	Group, in person	Trained facilitator	Art gallery	PTSD diagnosis
Lyshak-Stelzer et al.^60^, USA	29	RCT	Yes	TAU (unspecified)	16	16	UCLA PTSD-RI	North America (Amerian)	Art	Standalone	Group, in person	Licensed creative arts therapist	Inpatient unit	PTSD diagnosis
Momartin et al.^61^, Australia	32	Pre–post	No	N/A	30	36	SDQ	Mixed (mixed)	Dance	Standalone	Group, in person	Trained facilitator	School	Trauma symptoms
Moosa et al.^62^, India	30	Pre–post	No	N/A	5	3	DASS-21	Asia (India)	Art	Standalone	Group, in person	Trained facilitator	Refugee camp	Trauma symptoms
O’Callaghan et al.^63^, Ireland	159	RCT	Yes	Waitlist control	8	8	CRIES-8	Africa (Congolese)	Art	Standalone	Group, in person	Trained facilitator	Resident villages	PTSD symptoms
Pifalo^64^, USA	13	Pre–post	No	N/A	10	10	TSCC	North America (American)	Art	Standalone	Group, in person	Trained facilitator	Community center	PTSD symptoms
Pifalo^65^, USA	41	Pre–post	No	N/A	8	8	TSCC	North America (American)	Art	Standalone	Group, in person	Trained facilitator	Community center	PTSD symptoms
Pretorius and Pfeifer^66^, South Africa	25	Pre–post	No	N/A	8	8	TSCC	Africa (South African)	Art	Standalone	Group, in person	Trained facilitator	N.R.	Trauma symptoms
Quinlan et al.^67^, Australia	42	Pre–post	No	N/A	10+	10	HSCL-25	Mixed (mixed)	Art, music	Combined	Group, in person	Licensed creative arts therapist	School	Trauma symptoms
Staples et al.^68^, USA	129	Pre–post	No	N/A	N.R	10	CPSS	Middle East (Palestenian)	Mix of creative expression	Combined	Group, in person	Trained facilitator	Nonprofit organizations where participants were recruited	PTSD diagnosis
Thabet et al.^69^, Palestine	89	Pre–post	No	N/A	7	7	CPTSD-RI	Middle East (Palestenian)	Storytelling, play, art	Combined	Group, in person	Trained facilitator	Summer camp for refugee children	PTSD diagnosis
Tol et al.^70^, Indonesia	403	RCT	Yes	Waitlist control	15	5	CPSS	Asia (Indonesian)	Play, creative expression	Combined	Group, in person	Trained facilitator	School	PTSD symptoms
Truppi^71^, USA	28	Pre–post	No	N/A	N.R.	10	TSCC	North America (American)	Dance	Standalone	Group, in person	Licensed creative arts therapist	Hospital	Trauma symptoms
Ugurlu et al.^72^, Turkey	35	Pre–post	No	N/A	15	0.71	UCLA PTSD-RI	Middle East (Syrian)	Art, dance, music	Combined	Group, in person	Trained facilitator	Refugee houses	PTSD symptoms
van Westrhenen et al.^73^, South Africa	47	Pre–post	No	N/A	10	10	C-PTSD-C	Africa (South African)	Art, dance, music, drama, storytelling	Combined	Group, in person	Trained facilitator	Child abuse clinic	Trauma symptoms
Woollett et al.^74^, South Africa and USA	9	Pre–post	No	N/A	12	12	PTSD-RI	Mixed (mixed)	Art, play	Combined	Group, in person	Trained facilitator	Domestic violence shelter	PTSD symptoms
Zhang et al.^75^, Nigeria	470	RCT	Yes	TAU (unspecified)	6	6	ITQ	Africa (Nigerian)	Music, art or poetry	Standalone	Group, in person, interactive media	Licensed creative arts therapist	School	PTSD symptoms

CPSS, Child PTSD Symptom Scale for DSM-5; C-PTSD-C, Child PTSD Checklist; CPTSD-RI, Child Post-Traumatic Stress Disorder Reaction Index; CRIES-8, Children’s Revised Impact of Event Scale; CROPS, Child Report of Post-traumatic Symptoms; CRTES, Child’s Reaction to Traumatic Events Scale; DASS-21, The Depression, Anxiety and Stress Scale; HSCL-25, Hopkins Symptom Checklist-25; HTQ, Harvard Trauma Questionnaire; ITQ, International Trauma Questionnaire; N/A, not applicable; N.R., none reported; RCT, randomized controlled trial; TAU, treatment as usual; SDQ, Strength and Difficulties Questionnaire; PSS, PTSD Symptom Scale; PTSD-RI, UCLA Child/Adolescent PTSD Reaction Index for DSM-5; TSCC, Trauma Symptom Checklist for Children.

### Effectiveness of creative arts-based interventions among children and adolescents

**Fig. 2:**
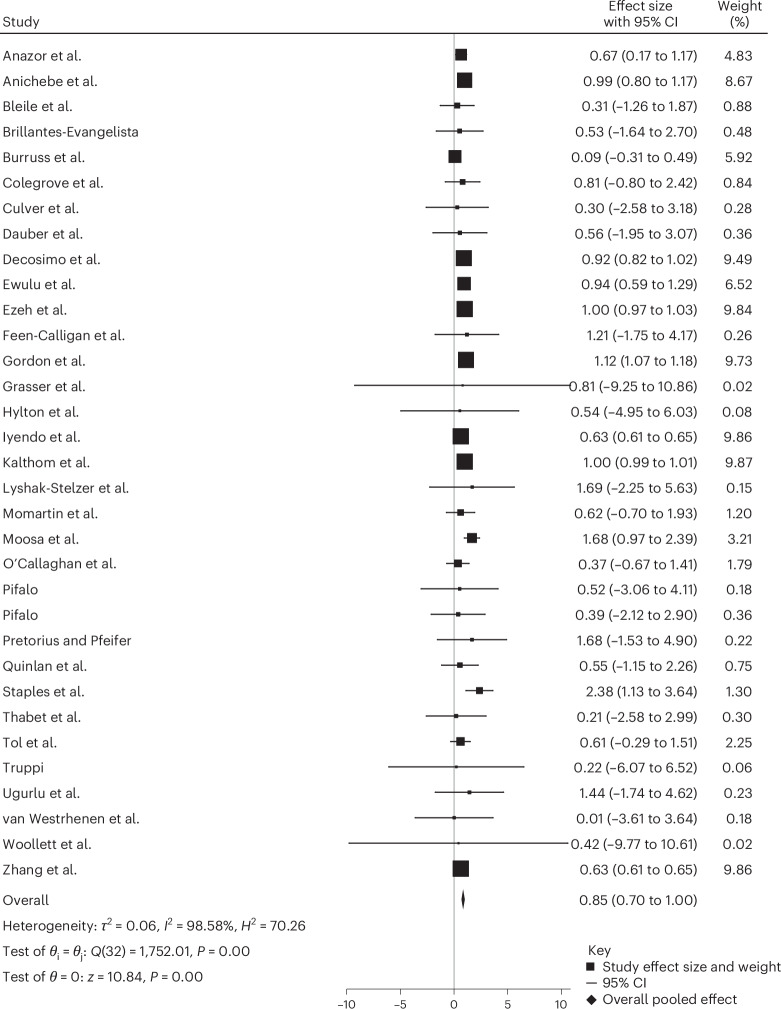
Forest plot of individual study effect sizes and pooled estimate. Effect sizes (*θ*ᵢ individual study Hedges’ *g*) and their 95% CIs were calculated for each study. The pooled effect size (*θ*) was estimated using a random-effects model with restricted maximum likelihood (REML). Heterogeneity across studies was evaluated using Cochran’s *Q* test (*Q*(32) = 1,752.01, *P* < 0.001) which tests the null hypothesis that all study effects are equal (*θ*ᵢ = *θ*ⱼ). The *I*² statistic (*I*² = 98.58%), *H*^2^ = 70.26 and *τ*² = 0.06. Further quantify the magnitude of between-study variability beyond chance. The overall pooled effect was statistically significant (*z* = 10.84, *P* < 0.001), with an estimated effect size of 0.85 (95% CI 0.70 to 1.00). All tests were two-sided. No adjustments for multiple comparisons were applied as this analysis addressed a single pooled hypothesis test.

The pooled effect size, reported as Hedges’ *g*, of the meta-analysis across all study types for the effect of creative arts-based interventions on PTSD symptoms are shown in Fig. 2. Effects are classified as small (0.2–0.5), medium (0.5–0.8) and large (≥0.08). All 33 studies^21,37,45–76^ included uncontrolled pre–post measurement of trauma or PTSD. The results of the pooled standardized effect sizes show a significant decrease in PTSD symptoms when a creative arts-based intervention was utilized with a large effect size (Hedge’s *g* of 0.85; *P* < 0.001; 95% confidence interval (CI) 0.70 to 1.00).

### Pre–post comparisons in nonrandomized trials

**Fig. 3:**
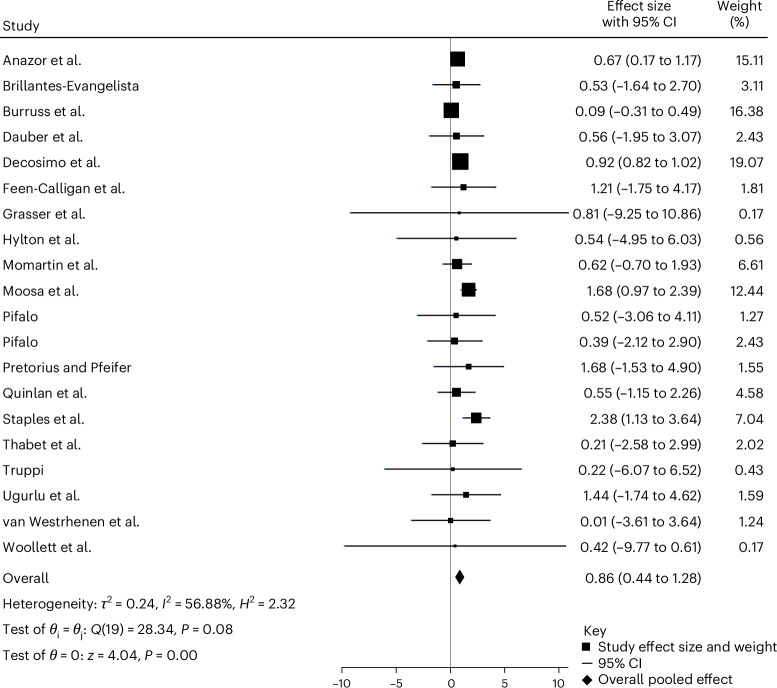
Forest plot of individual study effect sizes and pooled estimate. Effect sizes (Hedges’ *g*) and their 95% CIs were calculated for each study. The pooled effect size was estimated using a random-effects model with REML. Heterogeneity was assessed using Cochran’s *Q* test (*Q*(19) = 28.34, *P* = 0.08), the *I*² statistic (*I*² = 56.88%) and *τ*² = 0.24. The overall pooled effect was statistically significant (*z* = 4.04, *P* < 0.001) with an estimated effect size of 0.86 (95% CI 0.44 to 1.28). All tests were two-sided. No adjustments for multiple comparisons were applied as this analysis addressed a single pooled hypothesis test.

Data from a total of 20 nonrandomized studies^21,45,48,49,52,53,55,57,61,62,64–69,71–74^ using a sample of 1,442 participants at baseline to follow-up were included in this meta-analysis (Table 1). All 20 studies used uncontrolled pre–post designs and took PTSD measurements at baseline and follow-up, and 9 studies^45,47,48,54,66,67,69,71,73^ included nonrandomized comparison groups. However, in these studies, participants were not randomized to either intervention, which suggests the potential presence of selection bias. Nonetheless, the data suggest a significant reduction in PTSD symptoms from baseline to follow-up with an overall large effect size (Hedges’ *g* = 0.86; *P* < 0.001; 95% CI 0.44 to 1.28; Fig. 3).

### Controlled comparisons with active versus passive control groups

**Fig. 4:**
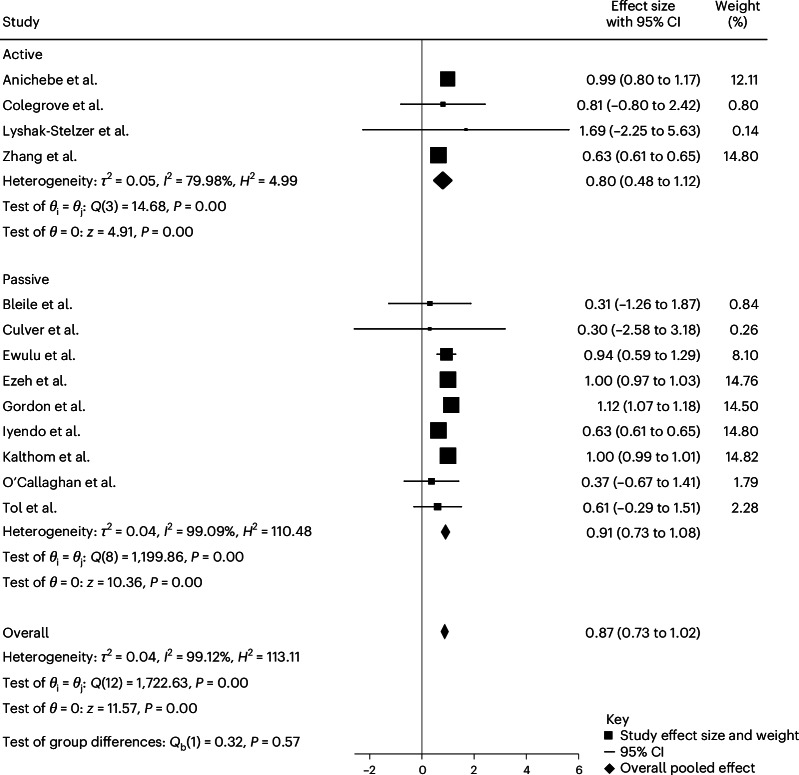
Subgroup meta-analysis of active versus passive interventions. The effect sizes (Hedges’ *g*) with 95% CIs were estimated using a random-effects REML model. Active interventions showed a significant pooled effect (*g* = 0.80; 95% CI 0.48 to 1.12; *Q*(3) = 14.68; *P* < 0.001; and *I*² = 79.98%), as did passive interventions (*g* = 0.91; 95% CI 0.73 to 1.08; *Q*(8) = 1,199.86; *P* < 0.001; and *I*² = 99.09%). The overall effect was significant (*g* = 0.87; 95% CI 0.73 to 1.02; *Q*(12) = 1,722.63; *P* < 0.001; *I*² = 99.12%). No subgroup difference was observed (*Q*_b_(1) = 0.32, *P* = 0.57). All tests two-sided with no adjustments for multiple comparisons.

We conducted a subgroup analysis on studies using controlled post-treatment comparisons with either an active or passive control condition. Four studies^46,50,60,75^ included an active control group (receiving treatment as usual, TAU) in addition to pre–post measurements (Fig. 4). Across the included studies, TAU was generally described by authors as routine care but was rarely specified in detail. It is unclear whether TAU involved evidence-based treatments such as CBT or EMDR, group therapy or nonspecific supportive care. Across these studies, 501 participants participated in a creative arts-based intervention and 362 in a control condition. At follow-up, those who participated in one of the creative arts-based interventions showed a lower PTSD score in comparison with those who participated in no intervention, continued treatment as usual or stayed on the waitlist. Results showed a significantly large effect size for the reduction of PTSD symptoms when a creative arts-based intervention was used compared to the active control condition TAU (Hedges’ *g* = 0.80; *P* < 0.001; and 95% CI 0.48 to 1.12).

Nine studies^37,47,51,54,56,58,59,63,70^ included a passive control group (no intervention and waitlist controls) in addition to pre–post measurements (Fig. 4). Across these studies, 1,142 participants participated in a creative arts-based intervention and 1,126 in a passive control condition. At follow-up, those who participated in one of the creative arts-based interventions showed a lower PTSD score in comparison with those who participated in no intervention or stayed on the waitlist. Results showed a significantly larger reduction of PTSD symptoms when a creative arts-based intervention was utilized compared to passive control conditions (Hedges’ *g* = 0.91; *P* < 0.001; 95% CI 0.73 to 1.08).

### Publication bias

All three meta-analyses showed high heterogeneity (75–95%) indicating the heterogeneity of the studies. The results of the Egger test indicated no significant evidence of small-study effects in the pre–post analysis, including all studies (*z* = −0.31 and *P* = 0.76), the nonrandomized pre–post interventions (*z* = 0.05 and *P* = 0.96) and those with active and passive control groups (*z* = −0.67 and *P* = 0.51), suggesting that publication bias is unlikely to have substantially influenced the meta-analysis results.

### Subgroup analysis of different types of creative arts-based interventions

**Fig. 5:**
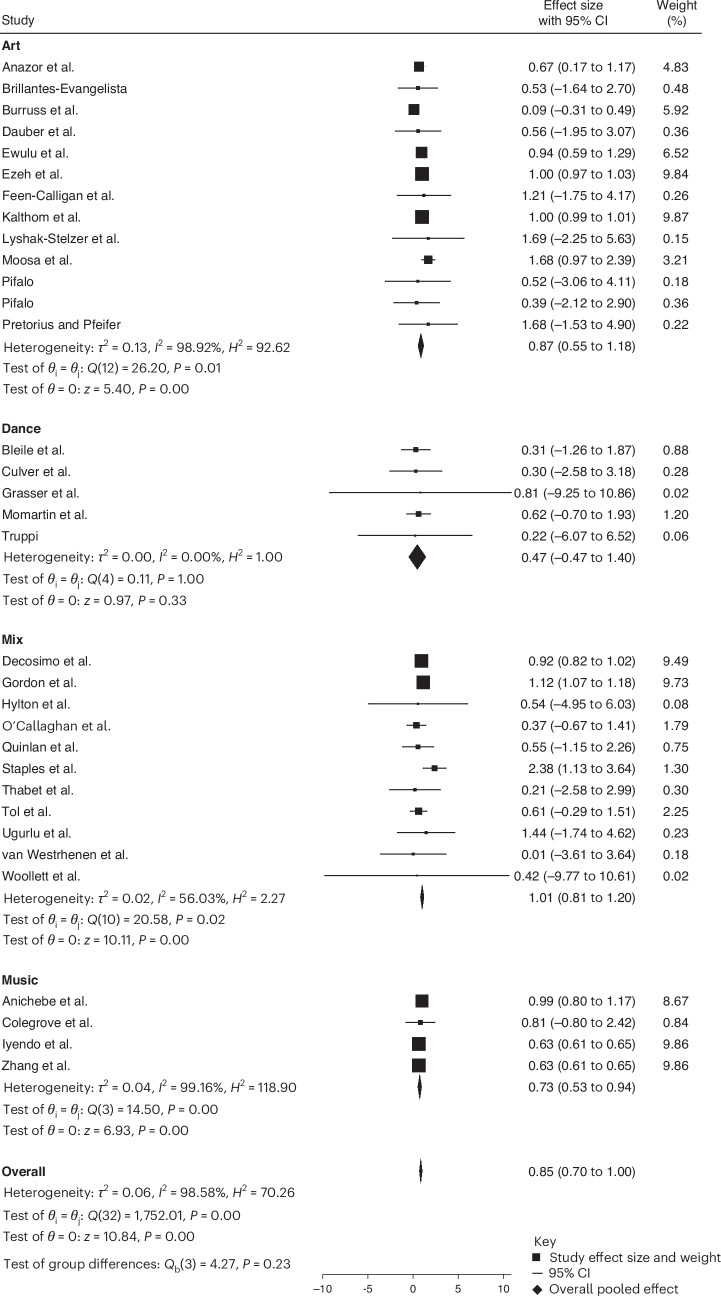
Subgroup meta-analysis by intervention type (art, dance, mixed and music). The effect sizes (Hedges’ *g*) with 95% CIs were estimated using a random-effects REML model. Pooled effects were: art (*g* = 0.79; 95% CI 0.56 to 1.03; *Q*(14) = 916.60; *P* < 0.001; *I*² = 99.14%), dance (*g* = 1.00; 95% CI 0.82 to 1.20; *Q*(5) = 13.86; *P* = 0.02; and *I*² = 0.00%), mixed (Mix) (*g* = 1.01; 95% CI 0.81 to 1.20; *Q*(10) = 20.58; *P* = 0.02; *I*² = 56.03%) and music (*g* = 0.63; 95% CI 0.61 to 0.65; *Q*(4) = 2.43; *P* = 0.66; *I*² = 0.04%). The overall pooled effect was significant (*g* = 0.83; 95% CI 0.70 to 0.95; *Q*(36) = 1,734.62, *P* < 0.001; *I*² = 98.25%). A significant subgroup difference was observed (*Q*_b_(3) = 271.36 and *P* < 0.001). All tests were two-sided with no adjustments for multiple comparisons.

A subgroup analysis was performed on the uncontrolled pre–post comparisons with the type of creative arts-based intervention. This subgroup analysis included both CAT interventions as well as creative arts-based interventions. Intervention types were only considered if they contained five or more studies (see Table 1 for a full breakdown). We thus considered art, dance, music and a mixed group that included creative arts-based interventions that utilized music, dance, art, drama and poetry in different sessions in combination. Results indicate a significant and large effect for mixed interventions (Hedges’ *g* = 1.01; *P* < 0.001; 95% CI 0.81 to 1.20) and a significant and large effect for both art interventions (Hedges’ *g* = 0.79; *P* < 0.001; 95% CI 0.56 to 1.03) and dance interventions (Hedges’ *g* = 1.00; *P* < 0.001; and 95% CI 0.96 to 1.04). In the music group, there was a significant medium effect for the reduction of PTSD scores (Hedges’ *g* = 0.63; *P* < 0.001; and 95% CI 0.61 to 0.65) (Fig. 5).

### Subgroup analysis of PTSD diagnostic status

We next performed an uncontrolled pre–post comparison subgroup analysis on PTSD diagnostic status in all 33 studies in three different categories: ‘PTSD diagnosis’, which represents studies where patients were diagnosed with PTSD based on the DSM-5 criteria^44^ before beginning the creative arts-based intervention; ‘PTSD symptoms’, which represents studies where patients had symptoms of PTSD but not a diagnosis; and ‘trauma symptoms’, which represents studies where patients have cited symptoms of trauma for a short period of time, but not a full presentation of PTSD symptoms or a PTSD diagnosis.

**Fig. 6:**
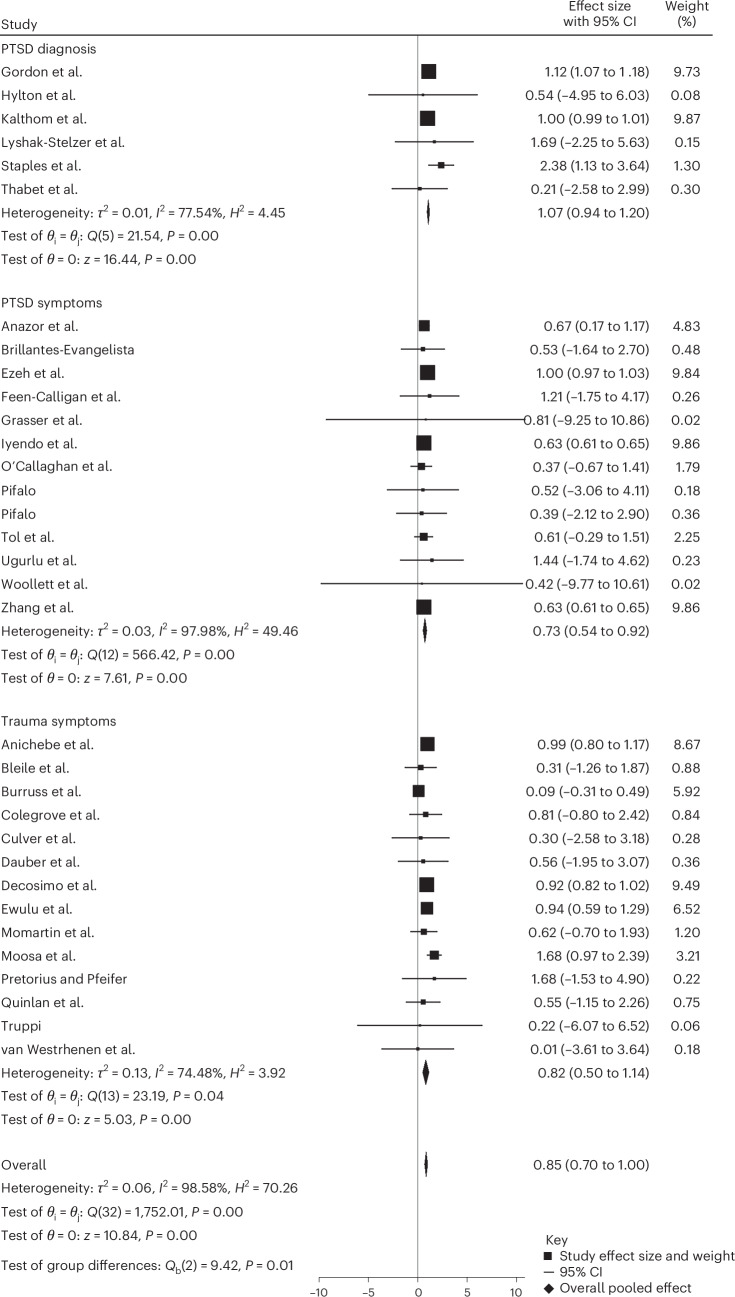
Subgroup meta-analysis by outcome type. Effect sizes (Hedges’ *g*) with 95% CIs were estimated using a random-effects REML model. Pooled effects were PTSD diagnosis (*g* = 1.07; 95% CI 0.94 to 1.20; *Q*(5) = 21.54; *P* < 0.001; *I*² = 77.54%), PTSD symptoms (*g* = 0.73; 95% CI 0.54 to 0.92; *Q*(12) = 566.42; *P* < 0.001; *I*² = 97.98%) and trauma symptoms (*g* = 0.82; 95% CI 0.50 to 1.14; *Q*(13) = 23.19; *P* = 0.04; *I*² = 74.48%). The overall pooled effect was significant (*g* = 0.85; 95% CI 0.70 to 1.00; *Q*(32) = 1,752.01; *P* < 0.001; *I*² = 98.58%). A test of subgroup differences was significant (*Q*_b_(2) = 9.42 and *P* = 0.01). All tests were two-sided with no adjustments for multiple comparisons.

This analysis yielded a significant reduction in PTSD and trauma symptoms with a large effect size in both the PTSD diagnosis group (Hedges’ *g* = 1.07; *P* < 0.0001; and 95% CI 0.94 to 1.20) and the trauma symptoms group (Hedges’ *g* = 0.82; *P* < .0001; 95% CI 0.50 to 1.14). Additionally, there was a medium effect size and a significant reduction in the PTSD symptoms group (Hedges’ *g* = 0.73; *P* < 0.001; 95% CI 0.54 to 0.92) (Fig. 6).

### Meta-regression analysis on duration of intervention

We conducted meta-regression analysis (uncontrolled and pre–post) on the continuous variable ‘time interval in weeks’ of the creative arts intervention. Overall, 31 studies^21,37,45,46,48,50–75^ (*n* = 4,048) reported the length of time of creative arts-based interventions. Results show that the effect of the time interval was not significantly associated with a reduction in PTSD scores over time (*β* = −0.006; s.e.m. = 0.011; *P* = 0.572; 95% CI −0.027 to 0.015). This means that the amount of time the creative arts-based intervention lasted did not significantly effect the reduction of PTSD scores in the patient population.

### Effectiveness of interventions by therapist qualification

To examine whether the effectiveness of creative arts-based interventions differed on the basis of the qualifications of the interventionist, we conducted a subgroup analysis in 32 studies with uncontrolled pre–post data to compare effects between interventions delivered by licensed creative arts therapists and those delivered by trained facilitators (one study was excluded as it contained a mixture of licensed creative therapists and trained facilitators^52^). The pooled effect size was significantly larger for interventions delivered by trained facilitators compared to licensed creative arts therapists, suggesting that trained facilitators may deliver creative arts-based interventions with comparable or even greater efficacy (Supplementary Fig. 4).

### Meta-regression analysis of within-study differences by intervention type

To further explore whether effect sizes differed by intervention type within studies, a series of random-effects meta-regressions were conducted on the six studies^37,46,48,54,58,75^ (uncontrolled and pre–post comparison) that included multiple CATs. Intervention type was included as a categorical moderator, and each model rotated the reference category (art, music, dance, drama and poetry) to enable all pairwise comparisons between modalities. Across all models, the meta-regression revealed no significant differences in effect sizes between intervention types (Wald *χ*²(4) = 2.24 and *P* = 0.692), and the moderator explained none of the between-study heterogeneity (*R*² = 0.00).

These findings suggest that, within studies directly comparing multiple creative arts-based interventions, no single modality (for example, art, music, dance, drama or poetry) showed a consistent advantage over another. The therapeutic benefit observed may therefore reflect a shared underlying mechanism common to CATs rather than being driven by one specific modality (Supplementary Table 3).

### Effectiveness of creative arts-based interventions among non-Western children and adolescents

To address our second research aim, we next compared the effectiveness of creative arts-based interventions in Western and non-Western populations by grouping the studies according to participants’ region of origin (for example, the Middle East). This subgroup analysis used uncontrolled pre–post comparisons within each region. Populations were only considered if they contained five or more studies. Thus, only samples from Africa, the Middle East and North America were analyzed for subgroup effects. Africa comprised mostly samples from Nigeria, the Middle East samples from Syria and Palestine, and North America samples from only the USA. While there were five studies in the mixed group, this group contained too many different ethnicities to draw meaningful conclusions.

**Fig. 7:**
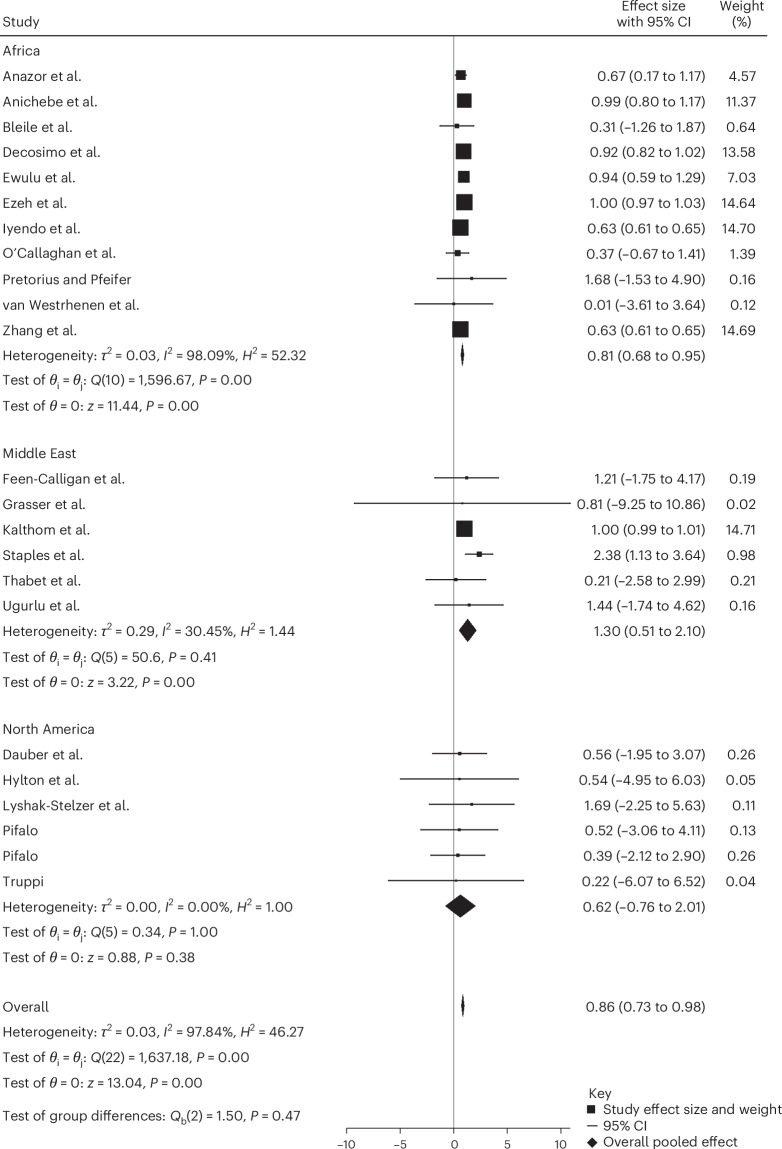
Subgroup meta-analysis by region. The effect sizes (Hedges’ *g*) with 95% CIs were estimated using a random-effects REML model. Pooled effects were Africa (*g* = 0.81; 95% CI 0.68 to 0.95; *Q*(10) = 596.67; *P* < 0.001; *I*² = 98.09%), the Middle East (*g* = 1.30; 95% CI 0.51 to 2.10; *Q*(5) = 5.06; *P* = 0.41; *I*² = 30.45%) and North America (*g* = 0.62; 95% CI –0.76 to 2.01; *Q*(5) = 0.34; *P* = 1.00; *I*² = 0.00%). The overall pooled effect was significant (*g* = 0.86; 95% CI 0.73 to 0.98; *Q*(22) = 1,637.18; *P* < 0.001; *I*² = 97.84%). No significant subgroup differences were observed (*Q*_b_(2) = 1.50 and *P* = 0.47). All tests were two-sided with no adjustments for multiple comparisons.

We found significant reductions in PTSD symptoms from pre- to post-creative arts-based interventions in Africa^37,45–47,53,54,58,63,66,73,75^ (*n* = 3,353; Hedges’ *g* = 0.81; *P* < 0.001; 95% CI 0.68 to 0.95) and the Middle East^21,55,59,68,69,72^ (*n* = 317; Hedges’ *g* = 1.30; *P* < 0.001; 95% CI 0.51 to 2.10) but not in North America^52,57,60,64,65,71^ (*n* = 176; Hedges’ *g* = 0.62; *P* > 0.05; 95% CI −0.76 to 2.01) (Fig. 7). In terms of effect sizes, the findings suggest a greater reduction in PTSD scores when a creative arts-based intervention was utilized within African and Middle Eastern samples; however, the differences between the three regions were not statistically significant.

## Discussion

This meta-analysis found that creative arts-based interventions significantly reduce PTSD symptoms in children and adolescents, with an overall large effect size (Hedges’ *g* = 0.85).

Subgroup analysis suggested that children with a PTSD diagnosis or trauma symptoms may benefit more from creative interventions. We also found that the duration of creative arts-based intervention was not associated with a reduction in PTSD scores over time, suggesting that the length of time an individual participates in a creative arts intervention does not impact the overall effect of their PTSD symptoms. As previous meta-analyses have not explored the effect of study duration on symptom reduction, follow-up analyses should be conducted to assess if the length of participation impacts symptom reduction as well as the duration of effects post-intervention.

A meta-regression of the six studies^37,46,48,54,58,75^ with multiple creative arts-based intervention types revealed no significant differences in effect sizes between CATs, suggesting that therapeutic benefits may be shared across modalities rather than specific to any one intervention type.

In terms of the creative arts intervention that produced the largest significant effect in the reduction of PTSD scores, mixed modalities (for example, music, art and dance) showed the strongest effects (Hedges’ *g* = 1.01), followed by dance (Hedges’ *g* = 1.00) and art (Hedges’ *g* = 0.79).

We also observed that interventions delivered by trained facilitators, not necessarily licensed creative arts therapists, were associated with slightly greater symptom reductions. While this finding should be interpreted cautiously, it highlights the potential for community-led, cost-effective models that increase accessibility without compromising impact.

Subgroup analyses revealed particularly large effects in non-Western samples, including West African and Middle Eastern youth. This may reflect both the cultural salience of creative expression and nonverbal accessibility in these populations. Creative arts interventions appeared to be more effective when participants were displaced from their country of origin (for example, Palestinian youth in the USA) perhaps owing to the added therapeutic value of cultural grounding in diasporic contexts. This supports the role of creative arts in enabling social engagement across cultures and emphasizes the need for cultural competence in delivery^77^.

By contrast, Western-based interventions in North America showed smaller and statistically nonsignificant effects. However, owing to mixed ethnicity in North American samples, it is difficult to tell whether creative arts-based interventions are generally less effective in North American children or less effective specifically in the racial majority compared to minoritized children. Owing to the lack of statistical significance between groups, we cannot confidently attribute differences in effect to region alone. More research is needed to explore how cultural, structural and contextual factors might shape the effectiveness of creative interventions across diverse settings.

Our findings build on previous reviews^42^, which reported only modest effects for creative therapies in youth. We observed larger effects, probably owing to stricter inclusion criteria and a more diverse sample base, including high-quality RCTs and non-Western contexts. Moreover, this is the first meta-analysis, to our knowledge, to explicitly examine cultural and regional variation in treatment outcomes.

## Clinical and cultural implications

In Western regions of the world, children and adolescents from minoritized groups are less likely to access or benefit from conventional psychiatric care and more likely to report negative experiences with it^19,20,78^. Despite growing calls for culturally responsive care, such as those outlined in the UK’s Improving Access to Psychological Therapies framework^79^, current adaptations largely focus on modifying talking therapies, which may not fully address the needs of these populations.

Our findings suggest that creative arts-based interventions offer a promising alternative, particularly for culturally diverse and underserved youth. These interventions harness nonverbal modes of expression, allowing participants to process trauma in ways that may feel more accessible and less stigmatizing. The effectiveness of these approaches is especially notable in African and Middle Eastern samples, where creative practices are deeply embedded in cultural life. A recent survey, for example, found that 72% of West African participants reported frequent engagement in creative activities tied to community, adaptation and expression, suggesting that arts-based therapies may naturally align with cultural coping strategies^76^. Additionally, recent literature has cited the common use of arts-based therapies for working with Middle Eastern populations and the positive effects that they have on reducing trauma symptoms^72^.

Clinically, these interventions may also target key trauma-related mechanisms. By engaging both sensory (nonverbal) and narrative (verbal) systems, CATs help reprocess traumatic memories in a more integrated and less distressing way^80^. This is supported by neuroimaging studies, which show enhanced connectivity between brain regions responsible for memory and emotion regulation during arts-based interventions^81^.

While previous reviews have also focused on children and adolescents^40–42^, few have explicitly addressed how developmental stage may influence the effectiveness of CATs. Young people are still developing emotional regulation, verbal processing skills and identity formation, which may make them more responsive to nonverbal, expressive and relational interventions^80^. For example, arts-based therapies provide embodied, sensory experiences that may be more developmentally appropriate than cognitively demanding approaches such as CBT. Our findings may reflect this developmental sensitivity, particularly given the large effects seen across diverse settings. Future research should investigate which modalities are most effective at different ages, for instance, whether younger children benefit more from visual arts or play-based methods, while adolescents may engage more with music, drama or dance.

Given their accessibility, cultural relevance and psychological impact, CATs should be considered not only as adjuncts, but as potentially central interventions in trauma-informed care for young people, particularly those from underrepresented backgrounds.

## Limitations

Despite the strengths of the study, there are several limitations. A main limitation of this meta-analysis was the heterogeneity of the studies with respect to the type of creative arts intervention. The methods used across studies (for example, RCT, cross-sectional and pre–post) also varied greatly, as well as the duration of the interventions. Additionally, owing to the limited number of studies in this meta-analysis, the subgroup analysis compared the effectiveness of creative arts-based interventions in Western, African and Middle Eastern samples but could not investigate its effectiveness in other regions of the world. Therefore, in further research, it would be beneficial to examine the effectiveness of CATs in other cultures to make firmer conclusions on their effectiveness in non-Western populations.

It is also important to note that only 10 out of 33 studies were statistically significant^37,45,46,53,54,56,58,59,62,75^. Most of these studies were RCTs with sample sizes ranging from 30 to 470 participants and at least five sessions of a creative arts-based intervention. These studies included art, drama, music, poetry and a mixture of creative interventions. Seven of these studies^37,45,46,53,54,58,75^ were studies with homogenous samples of Nigerian participants, which again emphasizes the effective use of creative arts-based interventions in West African populations. Future studies related to creative arts-based interventions for PTSD should therefore focus on RCT study designs with large sample sizes for reliable, statistically significant results.

We were also unable to comment on creative arts-based intervention effectiveness in comparison to current standardized treatments. Our findings point towards a large effect of creative arts-based interventions for the treatment of PTSD symptoms in children. This suggests that creative arts-based interventions are as effective as tf-CBT^82^, which also yields a large effect (Hedges’ *g* = 1.14) and is even more effective than group CBT^83^ (Hedges’ *g* = 0.70), EDMR^12^ (no significant difference) or pharmacological treatments^84^ (standardized mean difference of −0.63), which yield medium-sized effects. A key limitation is the lack of clarity surrounding TAU conditions across included studies. While several studies used TAU as a control, they often failed to specify whether it involved evidence-based psychotherapy, general counseling or minimal support. This variability in comparator conditions may influence effect sizes and limits the precision of cross-study comparisons. Ideally, future research should not only compare creative arts-based interventions with established treatments in RCTs but also provide detailed descriptions of TAU to enhance interpretability. Future studies should also prioritize the use of well-defined control groups, randomization and large sample sizes to minimize selection bias and establish more robust evidence for comparative effectiveness.

## Conclusions

The results of this meta-analysis suggest that creative arts-based interventions may contribute to a reduction in symptoms of trauma and PTSD in children and adolescents from Middle Eastern and West African groups, preliminarily indicating the potential uses of creative arts-based interventions as culturally competent therapeutic interventions. Given the high global rates of PTSD and the long-lasting effects of the disorder, these findings of reduced PTSD scores in children and adolescents may be particularly noteworthy for those suffering from the disorder.

Further research is necessary, particularly RCTs that have well-defined interventions; control groups utilizing CBT, EDMR or pharmacological interventions; and long-term follow-ups to recommend creative arts-based interventions to relieve symptoms in children and adolescents, as well as studies that examine creative arts-based interventions among African and Middle Eastern youth in Western contexts. However, this review provides promising evidence of their potential uses in clinical settings.

## Methods

This meta-analysis was conducted in accordance with the Preferred Reporting Items for Systematic Reviews and Meta-Analyses (PRISMA) guidelines^85^. The protocol was preregistered with PROSPERO (ID no. CRD42023389789).

### Eligibility criteria

Studies were included if they met the following criteria: participants had a mean age under 18 years and exhibited symptoms of PTSD (subclinical or clinically diagnosed on the basis of DSM-5) and interventions involved creative arts-based interventions, such as music, dance, poetry, drama or art either as standalone treatments or combined with other mediums. Both RCTs and nonrandomized pre–post intervention studies published in English were included, with interventions facilitated by licensed or formally trained therapists and lasting at least 1 day. Comparators included CBT, EMDR, TAU, waitlist controls or no intervention. The primary outcome measure was PTSD symptom reduction from baseline to post-intervention.

Studies were excluded if they were systematic reviews, meta-analyses, case studies, conference proceedings, abstracts, unpublished theses or published before 2000. Studies were also excluded if they did not assess PTSD or trauma symptoms at both baseline and post-intervention. Studies were excluded if they were yoga, martial arts, Pilates, Tai Chi, mindfulness-based interventions, relaxation techniques, aromatherapy, reflexology, or acupuncture or talking therapies not as comparators.

### Search strategy and study selection

A comprehensive literature search was conducted across PubMed, PsycINFO, Web of Science, ProQuest, CINAHL and Medline (OVID) for studies published between 2000 and 17 September 2025. Search terms included variations of ‘PTSD’, ‘post-traumatic stress’, ‘trauma’, ‘child’, ‘adolescent’, ‘youth’ and ‘creative arts therapy’, alongside specific terms for music, dance, drama, art and poetry therapy. Boolean operators were used to refine the search strategy.

Two independent reviewers (B.A. and B.D.) screened titles and abstracts using EndNote 20 and Microsoft Excel. Full texts of eligible studies were retrieved for further assessment. Duplicates were removed and any discrepancies in inclusion decisions were resolved by consulting a third reviewer (O.S.). Additional studies were identified through hand searching and citation chaining of reference lists. Authors also searched reference lists of previous reviews and meta-analysis^40–42^ on this topic. The PRISMA flow diagram (Supplementary Fig. 1) shows the study selection process.

#### Protocol deviations

This study was preregistered in PROSPERO (ID no. CRD42023389789). However, there were two deviations from the original protocol. First, while the preregistration listed Scopus and Embase as databases to be searched, these were ultimately not included owing to the overlap of results with other databases. Second, an inconsistency was identified in the preregistration regarding the inclusion of studies from 1990 or 2000 onward. In this meta-analysis, studies published from 2000 onwards were included, in alignment with the availability of more standardized PTSD diagnostic criteria. These deviations are documented for full transparency and adherence to PRISMA guidelines.

### Quality assessment

Study quality and risk of bias were evaluated using the Joanna Briggs Institute (JBI) critical appraisal tools^86^, applying the checklists specific to RCTs, quasi-experimental studies and analytical cross-sectional studies. Studies were required to meet at least 80% of the checklist criteria to be included in the meta-analysis. Two reviewers independently assessed studies, with a cutoff of 80% for inclusion. Given the challenges in blinding participants and facilitators in arts-based interventions, this criterion was not factored into the overall study quality assessment. There were no conflicting judgements of the study quality between the two reviewers. Full quality assessment details are reported in Supplementary Table 2.

### Data extraction

Data were extracted into Microsoft Excel 16.79 by B.A. and B.D. then cross-checked by O.S. Extracted data included study characteristics (author, year, location, sample size, age and ethnicity), intervention details (creative arts-based intervention type, session duration and PTSD measure) and comparison group details (pre- and post-intervention PTSD scores, means and standard deviations). The primary outcome measure was change in PTSD symptoms from baseline to post-intervention.

### Data synthesis and statistical analysis

The meta-analysis was conducted using STATA 18.5^87^. Hedges’ *g* was used to quantify effect sizes, classified as small (0.2–0.5), moderate (0.5–0.8) or large (>0.8) (ref. ^88^). A random-effects model (restricted maximum likelihood, REML) was used to account for between-study heterogeneity. Subgroup analyses were conducted on the basis of region of origin, creative arts-based intervention type and PTSD diagnostic status. Studies were included in subgroup analyses if at least four studies were available per category.

Heterogeneity was assessed using Higgins’ *I*^2^, with values ≥75% indicating high heterogeneity. Publication bias was examined through Egger’s test^89^, and funnel plots were generated using meta funnelplot in STATA 18.5. Publication bias was considered low if *P* > 0.05 and Egger’s intercept was close to zero.

Sensitivity analysis conducted using the metanif command in Stata. Sequentially omitting each study did not meaningfully alter the pooled estimate, which ranged from Hedges’ *g* = 0.72 to 0.90 (95% CI 0.71 to 0.91). The overall pooled effect remained *g* = 0.84 (95% CI 0.83 to 0.84), indicating that no individual study unduly influenced the results (Supplementary Fig. 5). In addition, analyses excluding studies rated below high quality on the JBI appraisal (≥80% threshold) were conducted, and the pooled effect size remained consistent. Finally, the impact of model specification was tested by comparing fixed-effect and random-effects models, with no substantive change in effect direction or magnitude observed.

### Quality assessment results

All 33 studies included in the meta-analysis met the JBI quality criteria, though blinding of participants and facilitators was generally infeasible. The full quality assessment details are reported in Supplementary Table 2.

### Reporting summary

Further information on research design is available in the Nature Portfolio Reporting Summary linked to this article.

## Supplementary information

Supplementary Information Supplementary Figs. 1–5 and Tables 2 and 3.

Reporting Summary

Supplementary Data 1 Extracted study-level of the passive versus active control meta-analysis.

Supplementary Data 2 Extracted study-level of the pre–post meta-analysis.

Supplementary Code 1 STATA analysis script.

## Source data

Source Data Fig. 1 Extracted study-level dataset containing all effect sizes (Hedges’ *g*), standard errors, confidence intervals and study weights used to generate all forest and funnel plots and statistical analyses in the manuscript. This dataset constitutes the source data for all figures and tables.

## Data Availability

The datasets generated and analyzed during the current study are available via the Open Science Framework repository at 10.17605/OSF.IO/KD8HM (ref. ^90^). The repository includes the extracted study-level dataset used for the meta-analysis (including study identifiers, effect sizes, confidence intervals and moderator codings). Source data underlying all figures (for example, forest plots and funnel plots) and tables presented in the manuscript are also provided in the repository.

## Code availability

The Stata code (.do file) used to conduct the meta-analysis is available via the Open Science Framework repository at 10.17605/OSF.IO/KD8HM (ref. ^90^).
