# Supplementary material for: Creative arts-based interventions for the improvement of PTSD symptoms in young people: a meta-analysis with a focus on non-Western populations
Source: Nat Ment Health. 2025 Nov 27;3(12):1616–32. doi: 10.1038/s44220-025-00543-y (PMC12705461; doi:10.1038/s44220-025-00543-y)
Supplement: Supplementary file 1 — Supplementary Figs. 1–5 and Tables 2 and 3. [file 44220_2025_543_MOESM1_ESM.pdf]

nature mental health

Supplementary information

https://doi.org/10.1038/s44220-025-00543-y

Creative arts-based interventions for the improvement of PTSD symptoms in young people: a meta-analysis with a focus on non-Western populations

In the format provided by the authors and unedited

Supplementary Materials

Table 2: Quality assessment of studies included in the meta-analysis (n = 33) using the Joanna Briggs Institute Checklists for Randomized Controlled Trials, Quasi-Experimental Studies, and Cross-Sectional Studies. Individual checklist items were rated as "Y" = Yes (criterion met), "N" = No (criterion not met), or "U" = Unclear. Questions were grouped into overarching domains—Selection bias, Performance bias, Detection bias, Attrition bias, and Reporting/Analysis bias—to summarise domain-level risk. The proportion of "Yes" responses was calculated for each study to provide an overall quality score. Studies meeting ≥ 80 % of applicable criteria were classified as low risk of bias, those meeting 60–79 % as moderate risk, and those meeting < 60 % as high risk.

Experimental Studies

Checklist for Randomized Controlled Trials

Study (Reference)	Q1 True randomization	Q2 Allocation concealed	Q3 Groups similar at baseline	Q4 Participants blinded	Q5 Therapists blinded	Q6 Groups treated identically	Q7 Outcome assessors blinded	Q8 Outcomes measured same way	Q9 Outcomes reliable	Q10 Follow-up complete / analysed	Q11 Analysed in assigned groups	Q12 Appropriate statistics	Q13 Design deviations accounted	% "Yes"	Overall RoB	Selection bias (Q1–Q3)	Performance bias (Q4–Q6)	Detection bias (Q7–Q9)	Attrition bias (Q10–Q11)	Reporting bias (Q12–Q13)
Anichebe et al. 2024 (Nigeria)	Y	Y	Y	N	N	Y	Y	Y	Y	Y	Y	Y	Y	84.60%	Low	Low	High	Low	Low	Low
Colegrove et al. 2018	Y	Y	Y	N	N	Y	Y	Y	Y	Y	Y	Y	Y	84.60%	Low	Low	High	Low	Low	Low
Culver et al. 2015	Y	Y	Y	N	Y	Y	Y	Y	Y	Y	Y	Y	Y	92.30%	Low	Low	Low	Low	Low	Low
Decosimo et al. 2019	Y	Y	Y	N	N	Y	Y	Y	Y	Y	Y	Y	Y	84.60%	Low	Low	High	Low	Low	Low
Ezeh et al. 2023	Y	Y	Y	N	N	Y	U	Y	Y	Y	Y	Y	Y	84.60%	Low	Low	High	Some concerns	Low	Low
Gordon et al. 2008	Y	Y	Y	N	Y	Y	U	Y	Y	Y	Y	Y	Y	92.30%	Low	Low	Low	Some concerns	Low	Low
Iyendo et al. 2024	Y	Y	Y	N	N	Y	U	Y	Y	Y	Y	Y	Y	84.60%	Low	Low	High	Some concerns	Low	Low
Kalthom et al. 2023	Y	Y	Y	N	U	Y	U	Y	Y	Y	Y	Y	Y	92.30%	Low	Low	Some concerns	Some concerns	Low	Low
Lyshak-Stelzer et al. 2007	Y	Y	Y	N	N	Y	U	Y	Y	Y	Y	Y	Y	84.60%	Low	Low	High	Some concerns	Low	Low
O'Callaghan et al. 2014	Y	Y	Y	N	Y	Y	Y	Y	Y	Y	Y	Y	Y	92.30%	Low	Low	Low	Low	Low	Low
Tol et al. 2008	Y	Y	Y	N	U	Y	N	Y	Y	Y	Y	Y	Y	84.60%	Low	Low	Some concerns	High	Low	Low
Zhang et al. 2023	Y	Y	Y	N	U	Y	U	Y	Y	Y	Y	Y	Y	92.30%	Low	Low	Some concerns	Some concerns	Low	Low

Checklist for Cross Sectional Studies

Study (Reference)	Q1 Inclusion criteria clearly defined	Q2 Subjects and setting described	Q3 Exposure measured validly	Q4 Objective criteria used	Q5 Confounders identified	Q6 Confounders addressed	Q7 Outcomes measured validly	Q8 Appropriate statistics	% "Yes"	Overall RoB	Selection bias (Q1–Q2)	Measurement bias (Q3–Q4)	Confounding bias (Q5–Q6)	Outcome/analysis bias (Q7–Q8)
Feen-Calligan et al. 2020	Y	Y	Y	Y	N	U	Y	Y	87.50%	Low	Low	Low	Some concerns	Low

Checklist for Quasi Experimental Studies

Study (Reference)	Q1 Cause–effect clear	Q2 Comparable participants	Q3 Similar care across groups	Q4 Control group present	Q5 Multiple measurements pre–post	Q6 Follow-up complete	Q7 Outcomes measured same way	Q8 Outcomes reliable	Q9 Appropriate statistics	% "Yes"	Overall RoB	Causality / Selection bias (Q1–Q2)	Performance bias (Q3–Q5)	Attrition bias (Q6)	Detection bias (Q7–Q8)	Reporting / Analysis bias (Q9)
Anazor et al. 2023	Y	Y	Y	Y	U	Y	Y	Y	Y	100%	Low	Low	Low	Some concerns	Low	Low
Bleile et al. 2024	Y	Y	Y	Y	Y	Y	Y	Y	Y	100%	Low	Low	Low	Low	Low	Low
Brillantes-Evangelista et al. 2013	Y	Y	Y	Y	Y	Y	Y	Y	Y	100%	Low	Low	Low	Low	Low	Low
Burruss et al. 2021	Y	Y	Y	N	Y	Y	Y	Y	Y	88.90%	Low	Low	Some concerns	Low	Low	Low
Dauber et al. 2015	Y	Y	Y	N	Y	Y	Y	Y	Y	88.90%	Low	Low	Some concerns	Low	Low	Low
Ewulu et al. 2024	Y	Y	Y	Y	Y	Y	Y	Y	Y	100%	Low	Low	Low	Low	Low	Low
Grasser et al. 2019	Y	Y	Y	N	Y	Y	Y	Y	Y	88.90%	Low	Low	Some concerns	Low	Low	Low
Hylton et al. 2019	Y	Y	Y	N	Y	Y	Y	Y	Y	88.90%	Low	Low	Some concerns	Low	Low	Low
Momartin et al. 2019	Y	Y	Y	N	Y	Y	Y	Y	Y	88.90%	Low	Low	Some concerns	Low	Low	Low
Moosa et al. 2017	Y	Y	Y	N	Y	Y	Y	Y	Y	88.90%	Low	Low	Some concerns	Low	Low	Low
Pifalo 2002	Y	Y	Y	N	Y	Y	Y	Y	Y	88.90%	Low	Low	Some concerns	Low	Low	Low
Pilao 2006	Y	Y	Y	N	Y	Y	Y	Y	Y	88.90%	Low	Low	Some concerns	Low	Low	Low
Pretorius & Pfeifer 2017	Y	Y	Y	Y	Y	U	Y	Y	Y	100%	Low	Low	Low	Some concerns	Low	Low
Quinlan et al. 2016	Y	Y	Y	Y	Y	N	Y	Y	Y	88.90%	Low	Low	Low	Some concerns	Low	Low
Staples et al. 2011	Y	Y	Y	N	Y	Y	Y	Y	Y	88.90%	Low	Low	Some concerns	Low	Low	Low
Thabet et al. 2005	Y	Y	Y	Y	Y	Y	Y	Y	Y	100%	Low	Low	Low	Low	Low	Low
Truppi 2001	Y	Y	Y	Y	Y	Y	Y	Y	Y	100%	Low	Low	Low	Low	Low	Low
Ugurlu et al. 2016	Y	Y	Y	N	Y	Y	Y	Y	Y	88.90%	Low	Low	Some concerns	Low	Low	Low
van Westrhenen et al. 2019	Y	Y	Y	Y	Y	Y	Y	Y	Y	100%	Low	Low	Low	Low	Low	Low
Woollett et al. 2020	Y	Y	Y	N	Y	Y	Y	Y	Y	88.90%	Low	Low	Some concerns	Low	Low	Low

Reference Category	Comparison Group	Coefficient (β)	Std. Error	95% CI	p-value
**Art**	Dance	0.029	0.44	[-0.834, 0.892]	0.947
	Drama	0.199	0.411	[-0.606, 1.004]	0.628
	Music	-0.012	0.148	[-0.301, 0.278]	0.936
	Poetry	-0.141	0.216	[-0.565, 0.282]	0.513
**Music**	Art	0.012	0.148	[-0.278, 0.301]	0.936
	Dance	0.24	0.217	[-0.186, 0.665]	0.269
	Drama	0.211	0.411	[-0.595, 1.017]	0.608
	Poetry	-0.13	0.218	[-0.556, 0.297]	0.552
**Dance**	Art	-0.029	0.44	[-0.892, 0.834]	0.947
	Drama	-0.029	0.44	[-0.892, 0.834]	0.947
	Music	-0.24	0.217	[-0.665, 0.186]	0.269
	Poetry	-0.369	0.268	[-0.895, 0.156]	0.168
**Poetry**	Art	0.141	0.216	[-0.282, 0.565]	0.513
	Dance	0.369	0.268	[-0.156, 0.895]	0.168
	Drama	0.34	0.44	[-0.523, 1.204]	0.44
	Music	0.13	0.218	[-0.296, 0.556]	0.552
**Drama**	Art	-0.199	0.411	[-1.004, 0.606]	0.628
	Dance	-0.24	0.217	[-0.665, 0.186]	0.269
	Music	-0.211	0.411	[-1.017, 0.595]	0.608
	Poetry	-0.34	0.44	[-1.204, 0.523]	0.44

Table 3: Meta-regression analysis of within-study differences by intervention type (n = 6 studies). Reported values are Hedge's g coefficients with corresponding standard errors (SE), 95% confidence intervals (CI), and p-values. Qb refers to the test of between-group heterogeneity.

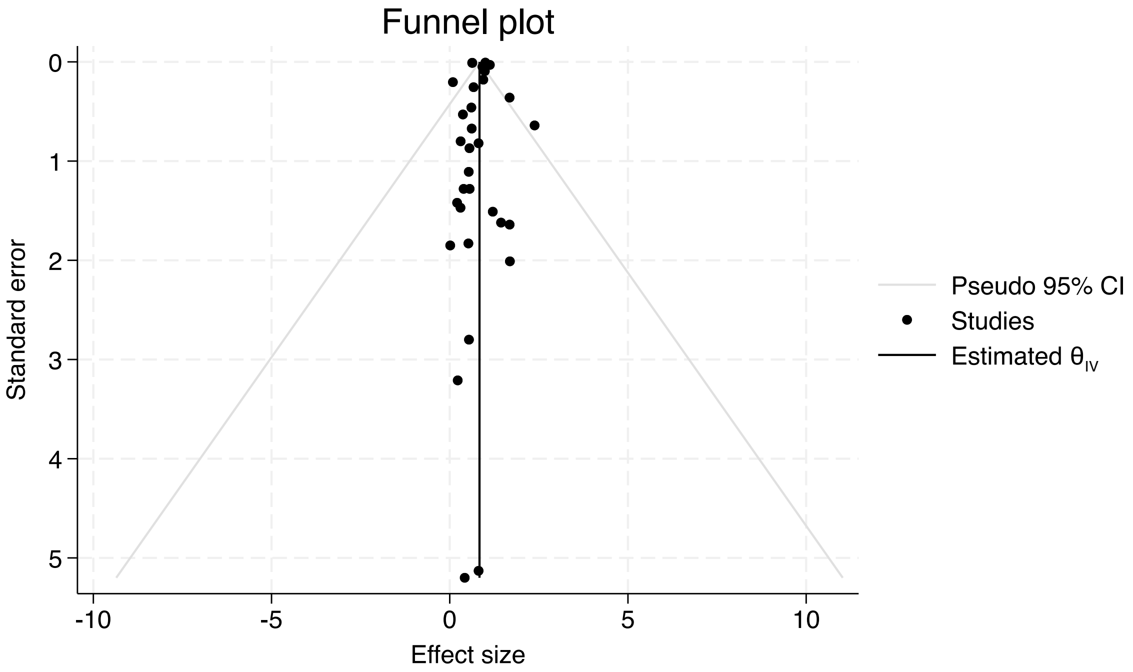

Supplementary Figure 1: Funnel plot of effect sizes from the pre-post meta-analysis (n = 33 studies). Each dot represents an individual study effect size plotted against its standard error. The vertical line indicates the pooled effect size from the random-effects model, and the grey triangle represents the pseudo 95% confidence limits. Egger's regression test for funnel plot asymmetry indicated no evidence of small-study effects (z = –0.31, p = 0.756).

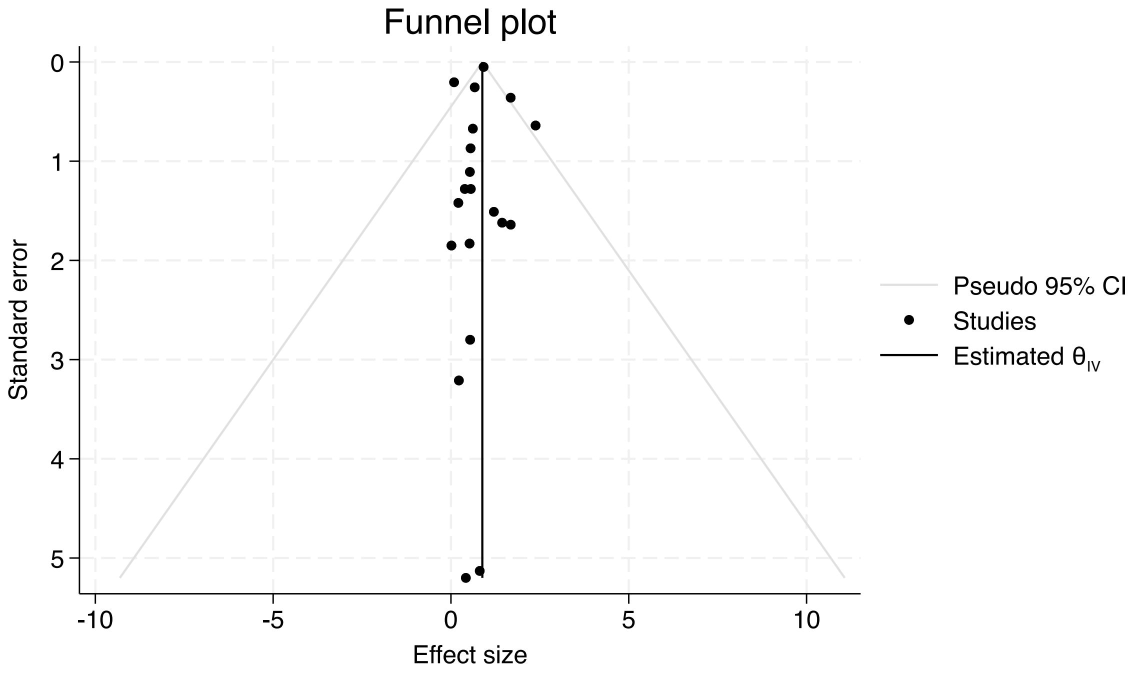

Supplementary Figure 2: Funnel plot of effect sizes from the pre-post meta-analysis (n = 20 studies). Each dot represents an individual study effect size plotted against its standard error. The vertical line indicates the pooled effect size from the random-effects model, and the grey triangle represents the pseudo 95% confidence limits. Egger's regression test for funnel plot asymmetry indicated no evidence of small-study effects (z = –0.05, p = 0.9566).

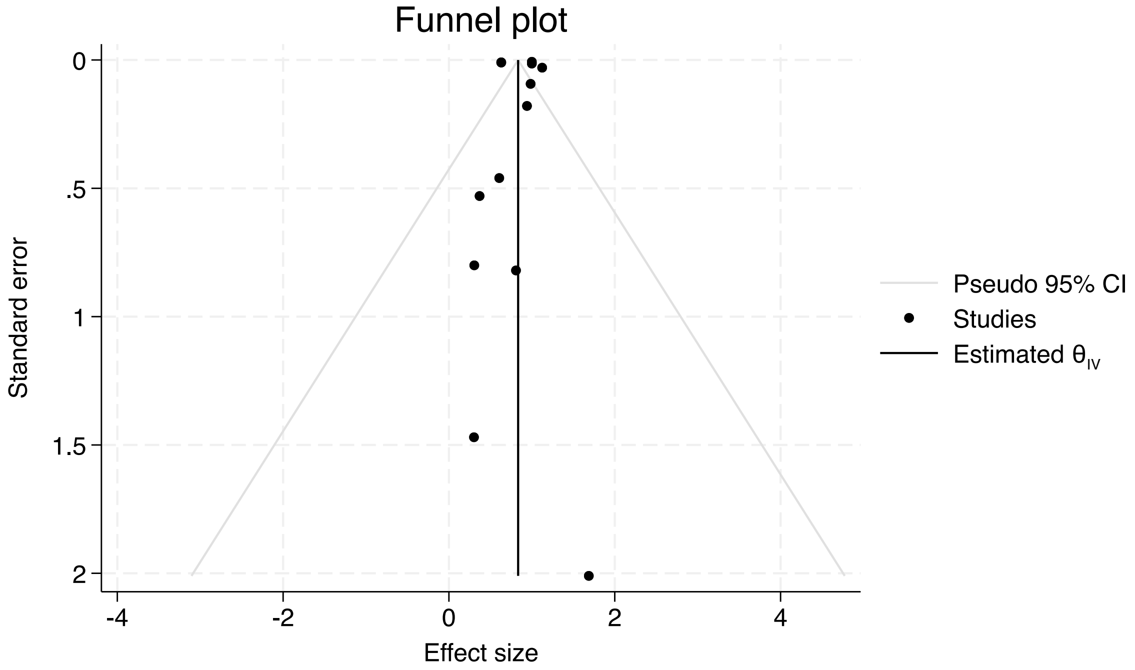

Supplementary Figure 3 Funnel plot of effect sizes from the pre-post meta-analysis (n = 13 studies). Each dot represents an individual study effect size plotted against its standard error. The vertical line indicates the pooled effect size from the random-effects model, and the grey triangle represents the pseudo 95% confidence limits. Egger's regression test for funnel plot asymmetry indicated no evidence of small-study effects (z = –0.67, p = 0.5052).

Effectiveness of Interventions by Therapist Qualification

To examine whether the effectiveness of creative arts-based interventions differed based on the qualifications of the interventionist, we conducted an uncontrolled, pre-post comparison subgroup analysis in 32 studies comparing interventions delivered by licensed creative arts therapists and those delivered by trained facilitators (one study was excluded as it contained a mixture of licensed creative therapists and trained facilitaors[51]). Licensed creative arts therapists typically possess graduate-level training and professional certification, while trained facilitators undergo more focused, intervention-specific training.

Both groups demonstrated significant reductions in PTSD symptoms from pre- to post-intervention. Interventions delivered by licensed creative arts therapists [21, 36, 44-45, 48-49, 52-54, 56-57, 59, 66, 70, 74] ($n = 2,307$, Hedge's $g = 0.76$, $p < 0.001$, 95% CI [0.59, 0.94]) and those delivered by trained facilitators[46-47, 50, 55, 58, 60-65, 67- 69, 71- 73] ($n = 1,678$, Hedge's $g = 1.06$, $p < 0.001$, 95% CI [0.94, 1.18]) were both effective in reducing PTSD symptoms. However, the pooled effect size was significantly larger for interventions delivered by trained facilitators compared to licensed creative arts therapists Qb = 7.28, $p = 0.01$), suggesting that trained facilitators may deliver Creative arts-based interventions with comparable or even greater efficacy.

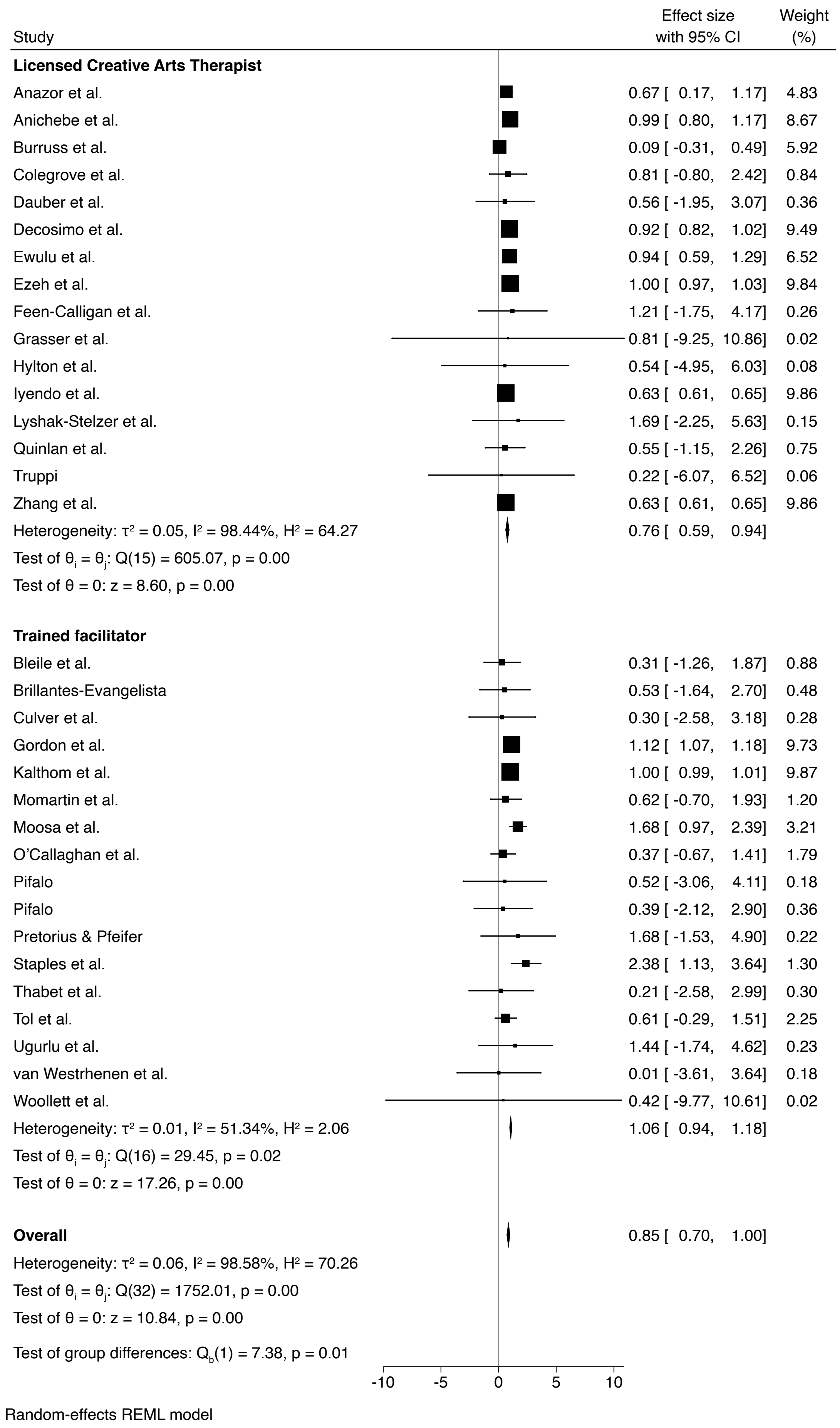
Study
Effect size with 95% CI
Weight (%)
Licensed Creative Arts Therapist
Anazor et al. 0.67 [0.17, 1.17] 4.83
Anichebe et al. 0.99 [0.80, 1.17] 8.67
Burruss et al. 0.09 [-0.31, 0.49] 5.92
Colegrove et al. 0.81 [-0.80, 2.42] 0.84
Dauber et al. 0.56 [-1.95, 3.07] 0.36
Decosimo et al. 0.92 [0.82, 1.02] 9.49
Ewulu et al. 0.94 [0.59, 1.29] 6.52
Ezeh et al. 1.00 [0.97, 1.03] 9.84
Feen-Calligan et al. 1.21 [-1.75, 4.17] 0.26
Grasser et al. 0.81 [-9.25, 10.86] 0.02
Hylton et al. 0.54 [-4.95, 6.03] 0.08
Iyendo et al. 0.63 [0.61, 0.65] 9.86
Lyshak-Stelzer et al. 1.69 [-2.25, 5.63] 0.15
Quinlan et al. 0.55 [-1.15, 2.26] 0.75
Truppi 0.22 [-6.07, 6.52] 0.06
Zhang et al. 0.63 [0.61, 0.65] 9.86
Heterogeneity: τ^2 = 0.05, I^2 = 98.44%, H^2 = 64.27 0.76 [0.59, 0.94]
Test of $\theta_i = \theta_j$: Q(15) = 605.07, p = 0.00
Test of θ = 0: z = 8.60, p = 0.00
Trained facilitator
Bleile et al. 0.31 [-1.26, 1.87] 0.88
Brillantes-Evangelista 0.53 [-1.64, 2.70] 0.48
Culver et al. 0.30 [-2.58, 3.18] 0.28
Gordon et al. 1.12 [1.07, 1.18] 9.73
Kalthom et al. 1.00 [0.99, 1.01] 9.87
Momartin et al. 0.62 [-0.70, 1.93] 1.20
Moosa et al. 1.68 [0.97, 2.39] 3.21
O'Callaghan et al. 0.37 [-0.67, 1.41] 1.79
Pifalo 0.52 [-3.06, 4.11] 0.18
Pifalo 0.39 [-2.12, 2.90] 0.36
Pretorius & Pfeifer 1.68 [-1.53, 4.90] 0.22
Staples et al. 2.38 [1.13, 3.64] 1.30
Thabet et al. 0.21 [-2.58, 2.99] 0.30
Tol et al. 0.61 [-0.29, 1.51] 2.25
Ugurlu et al. 1.44 [-1.74, 4.62] 0.23
van Westrhenen et al. 0.01 [-3.61, 3.64] 0.18
Woollett et al. 0.42 [-9.77, 10.61] 0.02
Heterogeneity: τ^2 = 0.01, I^2 = 51.34%, H^2 = 2.06 1.06 [0.94, 1.18]
Test of $\theta_i = \theta_j$: Q(16) = 29.45, p = 0.02
Test of θ = 0: z = 17.26, p = 0.00
Overall 0.85 [0.70, 1.00]
Heterogeneity: τ^2 = 0.06, I^2 = 98.58%, H^2 = 70.26
Test of $\theta_i = \theta_j$: Q(32) = 1752.01, p = 0.00
Test of θ = 0: z = 10.84, p = 0.00
Test of group differences: $Q_b(1)$ = 7.38, p = 0.01
-10
-5
0
5
10
Random-effects REML model

Supplementary Figure 4: Forest plot showing the uncontrolled pre–post effects of creative arts-based interventions on post-traumatic stress disorder (PTSD) symptoms, stratified by therapist qualification. Each square represents the effect size (Hedge's g) of an individual study, with square size proportional to its weight in the random-effects model. Horizontal lines denote 95% confidence intervals (CI), and diamonds represent pooled effect estimates for each subgroup and overall. n = 3,985 participants. Weights are from random-effects restricted maximum likelihood (REML) models. Between-group heterogeneity was significant (Q_b(1) = 7.38, p = 0.01).

Study omitted	Estimate	[95% Conf.	Interval]
Anazor et al.	.83528936	.82648605	.84409267
Anichebe et al.	.83488655	.82607436	.8436988
Bleile et al.	.83525443	.82645237	.84405655
Brillantes-Evangelista	.83524275	.82644069	.84404474
Burruss et al.	.83559984	.82679576	.84440392
Colegrove et al.	.83523858	.82643646	.84404063
Culver et al.	.83524275	.82644075	.84404474
Dauber et al.	.8352412	.82643914	.8440432
Decosimo et al.	.83455658	.82571888	.84339422
Ewulu et al.	.83517087	.82636613	.8439756
Ezeh et al.	.81633955	.80704647	.82563257
Feen-Calligan et al.	.83523446	.82643247	.84403646
Gordon et al.	.8285957	.81969345	.83749795
Grasser et al.	.8352378	.82643586	.84403974
Hylton et al.	.83523852	.82643658	.84404051
Iyendo et al.	.90294981	.89279306	.91310656
Kalthom et al.	.71998686	.70851219	.73146152
Lyshak-Stelzer et al.	.83523351	.82643157	.84403551
Momartin et al.	.83524758	.8264454	.84404969
Moosa et al.	.83510631	.82630366	.84390897
O'Callaghan et al.	.83527118	.82646888	.84407347
Pifalo	.83523965	.82643765	.84404165
Pifalo	.83524328	.82644123	.84404528
Pretorius & Pfeifer	.83523142	.82642943	.84403342
Quinlan et al.	.83524531	.82644325	.84404737
Staples et al.	.83516169	.82635951	.84396386
Thabet et al.	.83524406	.82644206	.84404606
Tol et al.	.83525956	.82645714	.84406191
Truppi	.83523899	.826437	.84404093
Ugurlu et al.	.83523315	.82643116	.84403515
van Westrhenen et al.	.83524263	.82644063	.84404463
Woollett et al.	.8352381	.8264361	.84404004
Zhang et al.	.88683438	.87698311	.8966856
Combined	.83523778	.82643582	.84403973

Supplementary Figure 5: Leave-one-out sensitivity analysis of creative arts-based interventions on PTSD symptoms. Each row shows the pooled effect size (Hedges' g) and its 95 % confidence interval (CI) after sequentially omitting one study from the meta-analysis. The vertical line represents the overall pooled effect from the complete model (g = 0.84, 95

% CI [0.83–0.84]). Across all iterations, pooled estimates remained highly stable (g = 0.72–0.90), indicating that no individual study substantially influenced the overall result. These findings confirm the robustness of the random-effects model (REML) and the consistency of the observed effect of creative arts-based interventions on PTSD symptom reduction.
