# Supplementary material for: Creative arts-based interventions for the improvement of PTSD symptoms in young people: a meta-analysis with a focus on non-Western populations
Source: Nat Ment Health. 2025 Nov 27;3(12):1616–32. doi: 10.1038/s44220-025-00543-y (PMC12705461; doi:10.1038/s44220-025-00543-y)
Supplement: Supplementary file 2 — Reporting Summary [file 44220_2025_543_MOESM2_ESM.pdf]

nature portfolio

Corresponding author(s): Ms Briana Applewhite

Last updated by author(s): 10/06/2025

Reporting Summary

Nature Portfolio wishes to improve the reproducibility of the work that we publish. This form provides structure for consistency and transparency in reporting. For further information on Nature Portfolio policies, see our Editorial Policies and the Editorial Policy Checklist.

Statistics

For all statistical analyses, confirm that the following items are present in the figure legend, table legend, main text, or Methods section.

n/a	Confirmed	
☐	☒	The exact sample size (n) for each experimental group/condition, given as a discrete number and unit of measurement
☐	☒	A statement on whether measurements were taken from distinct samples or whether the same sample was measured repeatedly
☐	☒	The statistical test(s) used AND whether they are one- or two-sided<br>*Only common tests should be described solely by name; describe more complex techniques in the Methods section.*
☐	☒	A description of all covariates tested
☒	☐	A description of any assumptions or corrections, such as tests of normality and adjustment for multiple comparisons
☐	☒	A full description of the statistical parameters including central tendency (e.g. means) or other basic estimates (e.g. regression coefficient) AND variation (e.g. standard deviation) or associated estimates of uncertainty (e.g. confidence intervals)
☐	☒	For null hypothesis testing, the test statistic (e.g. F, t, r) with confidence intervals, effect sizes, degrees of freedom and P value noted<br>*Give P values as exact values whenever suitable.*
☒	☐	For Bayesian analysis, information on the choice of priors and Markov chain Monte Carlo settings
☒	☐	For hierarchical and complex designs, identification of the appropriate level for tests and full reporting of outcomes
☐	☒	Estimates of effect sizes (e.g. Cohen's d, Pearson's r), indicating how they were calculated

Our web collection on statistics for biologists contains articles on many of the points above.

Software and code

Policy information about availability of computer code

Data collection

The data extraction was processed by B.A. into an electronic summary table using Endnote 20 and Microsoft Excel 16.79. The following data were extracted: Study and participant characteristics: author, study year, geographical location, sample size, average age, and ethnicity, study design (RCT/Intervention/Pre-Post); Intervention details: type CAT intervention, duration of treatment sessions, PTSD measure; Comparison details: pre and post-intervention PTSD score mean and standard deviation, control group PTSD score mean and standard deviation; Outcome measures: type of PTSD measure, Effect size (Hedge's g).
The principal endpoints were the change in mean PTSD scores from the baseline of the CAT intervention to after intervention, and control outcomes from the baseline to the end of intervention. Control group data was only extracted if the condition consisted of no intervention, treatment as usual (TAU) or individuals on the waitlist.

Data analysis

This meta-analysis was conducted using STATA 18.5. The effect size was extracted for each study along with the 95% confidence intervals (CI) using Hedge's g to quantify the intervention effect. he meta-analysis was conducted using the pre-post CAT intervention PTSD scores and a subgroup analysis was performed on the variables region of origin, CAT type, and PTSD symptom severity. For the purpose of this meta-analysis, if a region of origin contained four or more studies, it was deemed eligible for analysis.
All meta-analyses and sub-group analyses were conducted with random-effects models using the Dersimonian & Laird method. The Higgins I2 metric was used to estimate study heterogeneity. Heterogeneity was considered to be high when $I2 \geq 75\%$. The threshold for statistical significance for all analyses was $p < 0.05$. Publication bias was estimated with the Egger's test75 for small study effects, and funnel plots were created using the "meta funnelplot" command in STATA 18.5. Publication bias was considered to be low when $p > 0.05$ and Egger's intercept fell close to 0. The Duval and Tweedie trim and fill method was used to identify smaller studies causing funnel plot asymmetry and adjust for this asymmetry by imputing missing studies and re-estimating effect sizes.

For manuscripts utilizing custom algorithms or software that are central to the research but not yet described in published literature, software must be made available to editors and reviewers. We strongly encourage code deposition in a community repository (e.g. GitHub). See the Nature Portfolio guidelines for submitting code & software for further information.

Data

Policy information about availability of data

All manuscripts must include a data availability statement. This statement should provide the following information, where applicable:

- Accession codes, unique identifiers, or web links for publicly available datasets
- A description of any restrictions on data availability
- For clinical datasets or third party data, please ensure that the statement adheres to our policy

The author confirms that all data generated or analysed during this study are included in this published article. The datasets generated and analysed during the current study are openly available in the Open Science Framework (OSF) repository at DOI 10.17605/OSF.IO/KD8HM. The repository includes the extracted study-level dataset used for the meta-analysis (including study identifiers, effect sizes, confidence intervals, and moderator codings). Source data underlying all figures (e.g., forest plots, funnel plots) and tables presented in the manuscript are also provided in the repository. The Stata code (.do file) used to conduct the meta-analysis is openly available in the Open Science Framework (OSF) repository at DOI 10.17605/OSF.IO/KD8HM.

Research involving human participants, their data, or biological material

Policy information about studies with human participants or human data. See also policy information about sex, gender (identity/presentation), and sexual orientation and race, ethnicity and racism.

Reporting on sex and gender

Sex and gender data were not collected due to the inconsistencies in reporting this data throughout the 33 studies used within the meta-analysis.

Reporting on race, ethnicity, or other socially relevant groupings

Nationality data was collected and reported within this meta-analysis. One of the main aims of the analysis was to examine whether creative arts therapies are more effective among non-Western than Western youth.

Population characteristics

See above

Recruitment

As this is a meta-analysis of 33 studies, there were a variety of different ways of recruitment in each study. Studies recruited individuals from refugee camps, from clinical populations in nearby hospitals, from non-profit organisations offering 1+1 support and a multitude of other ways.

Ethics oversight

N/A

Note that full information on the approval of the study protocol must also be provided in the manuscript.

Field-specific reporting

Please select the one below that is the best fit for your research. If you are not sure, read the appropriate sections before making your selection.

☐ Life sciences ☒ Behavioural & social sciences ☐ Ecological, evolutionary & environmental sciences

For a reference copy of the document with all sections, see nature.com/documents/nr-reporting-summary-flat.pdf

Behavioural & social sciences study design

All studies must disclose on these points even when the disclosure is negative.

Study description

This meta-analysis of n=33 studies seeks to summarize existing results reported for creative arts-based interventions globally and provide novel insights into their potential applications for PTSD among youth in general and underrepresented (e.g., non-Western) youth in particular. All selected studies were quantitative with an experimental study design.

Research sample

The meta-analysis included a total pooled sample of 4,587 children and adolescents aged 3–18 years. Participants comprised both

Research sample	males and females and represented diverse cultural and geographic contexts across 15 countries, including studies conducted in Africa, the Middle East, Europe, North America, and Asia. The included studies encompassed a range of trauma exposures (e.g., war, displacement, abuse, and community violence) and intervention settings (schools, clinics, and community programs). The sample is considered broadly representative of children and adolescents affected by trauma globally, as studies were drawn from both Western and non-Western regions and from clinical as well as community populations.<br><br>The rationale for including this population was to capture the global variability in how creative arts-based interventions are implemented and to assess their effectiveness across culturally and contextually diverse youth populations. This inclusive approach allows for examination of whether intervention effects generalize beyond Western settings, where most mental health research has traditionally been conducted.
Sampling strategy	Two authors (B.A. and B.D) independently searched and screened the titles and abstracts of the articles up until the 17th of September 2025. to ascertain whether they fulfilled the inclusion criteria. B.A. conducted supplementary hand searching and citation-chaining by appraising the reference lists of selected articles. The full texts of relevant studies were retrieved and independently assessed for eligibility. All duplicate articles were removed from the final references. Search terms included variations of "PTSD," "post-traumatic stress," "trauma," "child," "adolescent," "youth," and "creative arts therapy", alongside specific terms for music, dance, drama, art, and poetry therapy. Boolean operators were used to refine the search strategy. Sample sizes of each of the studies was quite variable; studies included 9 to 500 participants in total. After screening 3602 references and reviewing 86 full-text articles, we included 33 studies (n=4,587).<br><br>Because this study is a systematic review and meta-analysis, no participants were directly recruited, and therefore no sampling procedure (e.g., random, stratified, or convenience sampling) was applied. The analytic sample comprised previously published studies that met predefined inclusion criteria following a systematic search of six databases. Eligible studies included randomized controlled trials, quasi-experimental studies, and analytical cross-sectional designs that quantitatively assessed PTSD symptoms before and after a creative arts-based intervention.<br><br>A formal sample size calculation was not conducted, as the meta-analysis was based on all available studies meeting the inclusion criteria at the time of search completion. The final dataset included 33 studies (n = 4,587 participants), which exceeds the minimum number typically required to achieve stable pooled estimates and enables subgroup and moderator analyses to be performed with sufficient statistical power.
Data collection	Data for this meta-analysis were collected through a systematic literature search of six electronic databases: PubMed, PsycINFO, Web of Science, ProQuest, CINAHL, and MEDLINE (OVID). Searches covered publications from January 2000 to 17 September 2025. Boolean operators were used to combine the following search terms: (PTSD OR posttraumatic stress OR post-traumatic stress OR trauma) AND (child* OR adolescent OR school OR youth*) AND (creative arts therapy OR music* OR music therapy OR dance OR dance therapy OR movement OR drama OR drama therapy OR art OR art therapy OR poetry OR poetry therapy)*. Two independent reviewers (B.A. and B.D.) screened all titles and abstracts to determine eligibility, followed by full-text review of studies meeting inclusion criteria. Supplementary hand-searching and citation-chaining were also performed to identify additional relevant studies. All duplicate records were removed before screening.<br><br>Data were extracted directly from published manuscripts using a standardized coding framework developed for this review. The data collection process did not involve interaction with participants, and therefore no additional individuals were present during data extraction. Because this study is a meta-analysis of previously published data, blinding of reviewers to study hypotheses or conditions was not applicable. However, screening and data extraction were conducted independently by two authors to minimize selection bias and ensure reproducibility.
Timing	Studies were included from January of 2000 until the 17th of September 2025.
Data exclusions	Studies were excluded based on the following conditions:<br>1. Study Design: (i) Systematic reviews; (ii) Meta-analyses; (iii) Case studies; (iv) Conference proceedings; (v) Abstracts; (vi) Unpublished theses; and<br>2. Publication date: (i) Studies published before the year 2000; and<br>3. Outcomes measured: (i) PTSD or symptoms of trauma not assessed at baseline and at least once after intervention.<br><br>A total of 3,602 records were screened after duplicates were removed. Of these, 86 full-text articles were assessed for eligibility, and 33 studies met all inclusion criteria and were included in the meta-analysis. Exclusion criteria were pre-established based on study design, intervention type, and relevance to PTSD outcomes. Specifically, 53 full-text articles were excluded for the following reasons: qualitative study design (n = 19), not PTSD-related (n = 8), not a creative arts-based intervention (n = 18), symptoms not quantitatively assessed (n = 3), systematic review or meta-analysis (n = 3), and case report (n = 2). No additional data were excluded after inclusion decisions were made. A summary of the selection process is provided in the PRISMA flow diagram (FIgure 1).
Non-participation	As this study was a systematic review and meta-analysis of previously published data, no human participants were directly recruited or involved in the research process. Consequently, there were no participant dropouts, refusals, or response rates to report.
Randomization	Thirteen studies were Randomized Control Trials so participants were randomized into an experimental or control group. The remaining 20 studies were Quasi Experimental so participants were allocated to a creative arts intervention and PTSD was measured at two time points, pre and post test.

Reporting for specific materials, systems and methods

We require information from authors about some types of materials, experimental systems and methods used in many studies. Here, indicate whether each material, system or method listed is relevant to your study. If you are not sure if a list item applies to your research, read the appropriate section before selecting a response.

Materials & experimental systems

n/a	Involved in the study
☒	☐ Antibodies
☒	☐ Eukaryotic cell lines
☒	☐ Palaeontology and archaeology
☒	☐ Animals and other organisms
☒	☐ Clinical data
☒	☐ Dual use research of concern
☒	☐ Plants

Methods

n/a	Involved in the study
☒	☐ ChIP-seq
☒	☐ Flow cytometry
☒	☐ MRI-based neuroimaging

Plants

Seed stocks	N/A
Novel plant genotypes	N/A
Authentication	N/A
