# Supplementary material for: Creative arts-based interventions for the improvement of PTSD symptoms in young people: a meta-analysis with a focus on non-Western populations
Source: Nat Ment Health. 2025 Nov 27;3(12):1616–32. doi: 10.1038/s44220-025-00543-y (PMC12705461; doi:10.1038/s44220-025-00543-y)
Supplement: Supplementary file 5 — STATA analysis script. [file 44220_2025_543_MOESM5_ESM.pdf]

****

Project: **Creative arts-based Interventions for the improvement of PTSD symptoms in young people: a meta-analysis with a focus on non-Western populations**

Purpose: Reproduce meta-analysis reported in manuscript

Author: Briana Applewhite, Brennan Delattre, Ilina Singh, Morten Kringelbach,& Olivia Spiegler

Date: 01.10.2025

Software: Stata 18.5

Notes:

- This .do file contains the exact Stata commands used to generate the
  results (pooled analyses, subgroup analyses, meta-regressions, and plots).
- Dataset variables assumed:
    ES (effect size), SE (standard error), Author, Study_ID,
    Controltype, Interventiontype, Symptomseverity, Region, weeks

***/

```
*------------------------------------------------------
* 1) Overall forest plot (random effects, REML)
*------------------------------------------------------
meta forestplot, random(reml)


*------------------------------------------------------
* 2) Subgroup analyses (pooled results)
*------------------------------------------------------
meta summarize, random(reml) subgroup(Controltype)
meta summarize, random(reml) subgroup(Interventiontype)
meta summarize, random(reml) subgroup(Symptomseverity)
```

```
meta summarize, random(reml) subgroup(Region)

*--------------------------------------------------------
* 3) Meta-regression (continuous moderator: weeks)
*--------------------------------------------------------
meta regress weeks, random(reml)

*--------------------------------------------------------
* 4) Subgroup-specific forest plots
*--------------------------------------------------------
meta forestplot, random(reml) subgroup(Controltype)
meta forestplot, random(reml) subgroup(Symptomseverity)
meta forestplot, random(reml) subgroup(Region)
meta forestplot, random(reml) subgroup(Interventiontype)

*--------------------------------------------------------
* 5) Funnel plot
*--------------------------------------------------------
meta funnelplot

*--------------------------------------------------------
* 6) Meta-regression with robust variance estimation (RVE)
*    Clusters defined by Study_ID
*--------------------------------------------------------
meta regress i.Interventiontype, rve(Study_ID)

************************************************************************
```

* End of analysis.do

****
